# Proteomic clustering reveals the kinetics of disease biomarkers in bovine and human models of post-traumatic osteoarthritis

**DOI:** 10.1016/j.ocarto.2021.100191

**Published:** 2021-06-10

**Authors:** Rebecca Mae Black, Yang Wang, André Struglics, Pilar Lorenzo, Susan Chubinskaya, Alan J. Grodzinsky, Patrik Önnerfjord

**Affiliations:** a Departments of Biological Engineering, Massachusetts Institute of Technology, Cambridge, MA, USA; b Orthopaedics, Department of Clinical Sciences Lund, Faculty of Medicine, Lund University, Lund, Sweden; c Rheumatology and Molecular Skeletal Biology, Department of Clinical Sciences Lund, Faculty of Medicine, Lund University, Lund, Sweden; d Departments of Pediatrics, Orthopedic Surgery and Medicine (Section of Rheumatology), Rush University Medical Center, Chicago, IL, USA; e Mechanical Engineering, Massachusetts Institute of Technology, Cambridge, MA, USA; f Electrical Engineering and Computer Science, Massachusetts Institute of Technology, Cambridge, MA, USA

**Keywords:** Post-traumatic osteoarthritis, Mass spectrometry, Cartilage matrix, Cytokines, Proteomics, Dexamethasone

## Abstract

**Objectives:**

In this study, we apply a clustering method to proteomic data sets from bovine and human models of post-traumatic osteoarthritis (PTOA) to distinguish clusters of proteins based on their kinetics of release from cartilage and examined these groups for PTOA biomarker candidates. We then quantified the effects of dexamethasone (Dex) on the kinetics of release of the cartilage media proteome.

**Design:**

Mass spectrometry was performed on sample medium collected from two separate experiments using juvenile bovine and human cartilage explants (3 samples/treatment condition) during 20- or 21-day treatment with inflammatory cytokines (TNF-α, IL-6, sIL-6R) with or without a single compressive mechanical injury. All samples were incubated with or without 100 ​nM Dex. Clustering was performed on the correlation between normalized averaged release vectors for each protein.

**Results:**

Our proteomic method identified the presence of distinct clusters of proteins based on the kinetics of their release over three weeks of culture. Clusters of proteins with peak release after one to two weeks had biomarker candidates with increased release compared to control. Dex rescued some of the changes in protein release kinetics the level of control, and in all conditions except control, there was late release of immune-related proteins.

**Conclusions:**

We demonstrate a clustering method applied to proteomic data sets to identify and validate biomarkers of early PTOA progression and explore the relationships between the release of spatially related matrix components. Dex restored the kinetics of release to many matrix components, but not all factors that contribute to cartilage homeostasis.

## 1 Introduction

There are over five million annual U.S. cases of posttraumatic osteoarthritis (PTOA), the degeneration of cartilage and subchondral bone after a traumatic joint injury. However, not all traumatic joint injuries progress to PTOA: reported PTOA prevalence after anterior cruciate ligament (ACL) rupture is only 13–40%, with higher prevalence if combined with a meniscal injury [1]. While there are no approved disease-modifying drugs for OA or PTOA, promising targets such as the corticosteroid dexamethasone (Dex) have been suggested to rescue cartilage matrix breakdown and prevent chondrocyte death in models of PTOA [[2], [3], [4], [5]] [[2], [3], [4], [5]] [[2], [3], [4], [5]], but use of these drugs remains controversial due to a lack of consensus on potential off-target effects. PTOA is an attractive target for the application of disease-modifying drugs, as the time of disease onset, the injury, is known. However, some patients will not progress to PTOA after injury; therefore, a prognostic biomarker to differentiate the most at-risk patients is desirable.

An ideal prognostic biomarker would identify patients at risk of PTOA before cartilage degeneration becomes irreversible. Previously identified biomarker candidates include matrix molecules indicative of cartilage catabolism – i.e. aggrecan, cartilage oligomeric matrix protein (COMP), collagen II (and associated degradation products such as crosslinked C-telopeptide collagen II fragments (CTX-II)); proinflammatory cytokines, e.g. tumor necrosis factor-alpha (TNF-α), interleukins (IL) −1, −6, −8, and −10; and proteases and protease inhibitors, e.g. matrix metalloproteinase (MMP) −3 and −13 and tissue inhibitor of metalloproteinase 1 (TIMP-1) [6,7]. Though there are many potential biomarker candidates identified in the literature, their prognostic use to predict the patients most at risk for PTOA still remains unclear [6]. In one study of synovial fluid aspirates after ACL injury, aggrecan, COMP, MMP-3, and TIMP-1 were elevated at early time points, but failed to predict radiographic knee OA 16 years after ACL injury [8]. This suggests that it may be necessary to discover a more targeted combination of specific disease progression-associated biomarkers that considers the timing of the appearance of biomarkers in synovial fluid. Proteomics is an excellent tool for biomarker identification, as it can be used to screen samples from clinical or *in vitro* PTOA models for hundreds of proteins that could be biomarker candidates. The timing of biomarker release must be considered as well, as the cellular response to injury and PTOA progression is time-dependent with distinct catabolic and anabolic phases [9].

In *ex vivo* human and animal models of PTOA, Dex has been shown to have protective effects against disease progression, but literature reports conflict regarding its safety to cartilage and chondrocyte health [2]. In an IL-1 challenge of human cartilage explants, the low dose of 100 ​nM Dex rescued glycosaminoglycan (GAG) loss and maintained chondrocyte viability [4]. This finding was supported in two further studies utilizing IL-6 and TNF-α-challenged human explant models of PTOA that reported Dex attenuated GAG loss and MMP-1 release [5,10]. However, some studies using high doses and long durations of Dex treatment reported negative effects on cartilage, from chondrocyte cell death to cartilage degeneration in explant and whole-animal models [[11], [12], [13]] [[11], [12], [13]] [[11], [12], [13]]. Our recent proteomic study using a juvenile bovine explant model of PTOA reported that Dex attenuated catabolic effects of mechanical injury and IL-6/TNF-α-challenge, but did not rescue suppressive effects on certain anabolic pathways [3]. Differences between ages, species, and dosing in the above models complicate interpretation of the safety of Dex use, necessitating investigation into the effects of Dex on matrix breakdown in human explant models and how those results compare to commonly used animal models of PTOA.

In the present study, we demonstrate a method of clustering proteomic data using an existing proteomic data set from a bovine cartilage explant PTOA model, and validate this method by comparison to a human knee explant model of PTOA. Our aims are (1) to distinguish distinct clusters of proteins having different profiles of release from cartilage, (2) to determine biomarker candidates based on their timing and relative amount released to explant medium (as a surrogate for synovial fluid), and (3) to quantify the effects of Dex on the kinetics of release of the cartilage medium proteome.

## 2 Materials and methods

### 2.1 Explant harvest and culture

**Fig. 1:**
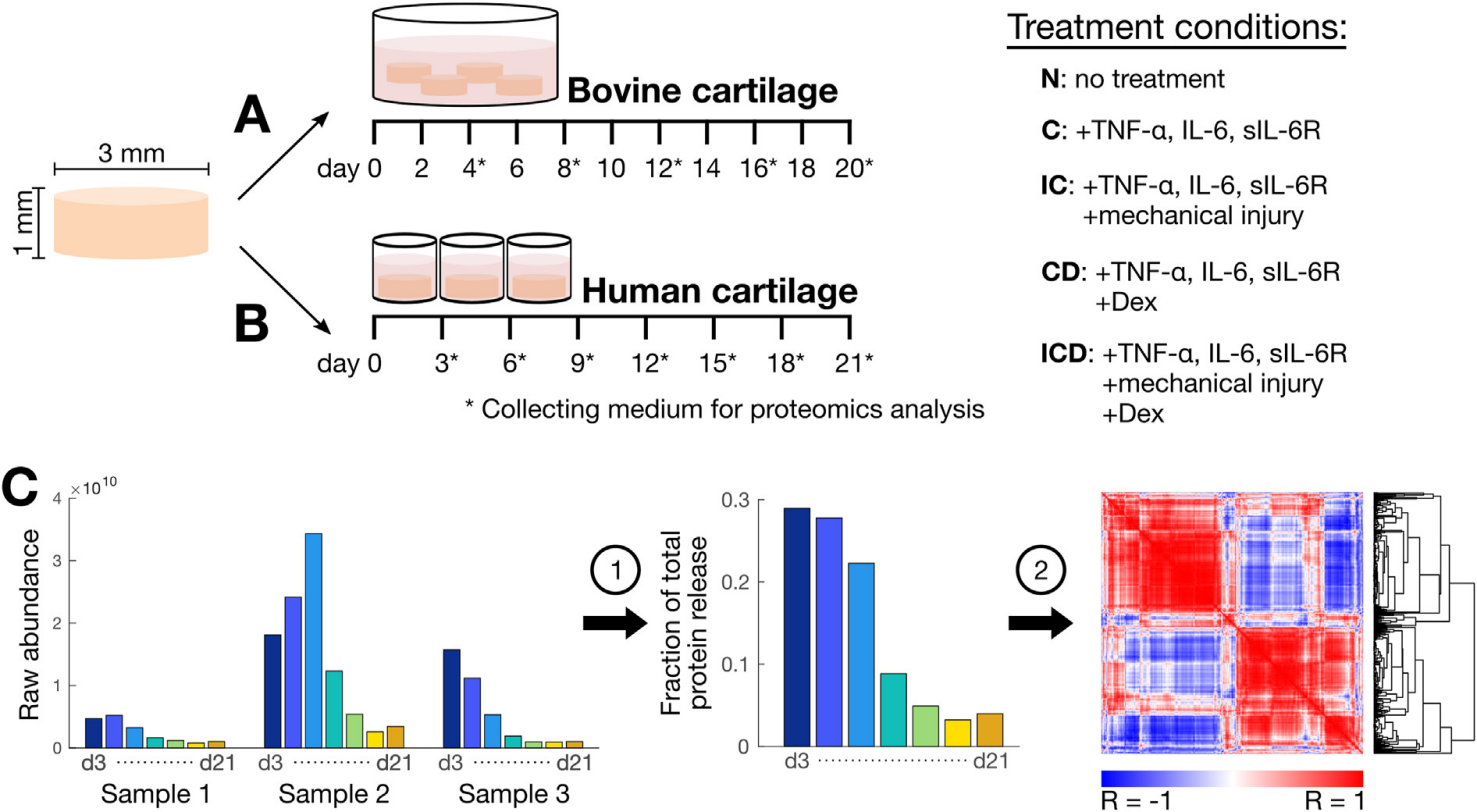
**Experimental overview and clustering method.** Bovine **(A)** and human **(B)** cartilage explants (3 ​mm diameter x 1 ​mm height) were cultured for 20 or 21 days with media collected every four or three days, respectively, for mass spectrometry analysis. The explants were untreated (N), treated with inflammatory cytokines (C, 10 ​ng/mL TNF-α ​+ ​20 ​ng/mL IL-6 ​+ ​100 ​ng/mL sIL-6R for bovine, 100 ​ng/mL TNF-α ​+ ​50 ​ng/mL IL-6 ​+ ​250 ​ng/mL sIL-6R for human), treated with cytokines plus a single impact mechanical injury (IC, 50% final strain at 100%/s strain rate for bovine, 60% final strain at 300%/s strain rate for human) or received treatments C and IC with 100 ​nM Dex (CD and ICD, respectively). **(C)** To cluster the proteins based on time release profile, the raw abundance data of each protein (represented by collagen II, COL2A1) was normalized to the total amount released within that replicate and averaged across all three replicates (1), then the resulting vectors for each protein were clustered based on correlation using Euclidian distance (2), which can be visualized in an n-by-n heatmap, where n is the number of proteins, and each comparison is colored based on correlation. R: correlation coefficient.

Proteomic data were obtained from two separate experiments: a juvenile bovine explant study [3] to establish the analytical technique, and a human data set [5] to validate the model. Cartilage disks (3 ​mm diameter x 1 ​mm height, including the intact superficial zone) were harvested from the femoropatellar grooves of three 1-2-week-old bovines (Research ’87, Boylston, MA) as described [4] (Fig. 1A). Human explants were harvested from the tibial plateau of a 74-year-old male donor (Collins Grade 1, near-normal tissue [14]) obtained postmortem through the Gift of Hope Organ and Tissue Donor Network (Itasca, IL) (Fig. 1B). All procedures were approved by Rush University Medical IRB and the MIT COUHES committee. After harvesting, explant disks were pre-equilibrated for two days. Details on media composition can be found in Supplemental Methods. Four bovine explants were cultured per well, with one replicate per treatment condition for each animal; human explants were cultured with one per well, and three total replicates per treatment condition (Fig. 1).

### 2.2 Explant treatments

After pre-equilibration, samples were treated for 20 or 21 days (Fig. 1A–B): no treatment (N), continuous culture with inflammatory cytokines (10 ​ng/mL recombinant human TNF-α, 20 ​ng/mL recombinant human IL-6 and 100 ​ng/mL soluble IL-6 receptor (sIL-6R) (R&D Systems) for bovine, 100 ​ng/mL TNF-α, 50 ​ng/mL IL-6, 250 ​ng/mL sIL-6R for human; treatment C), cytokines with a single mechanical impact injury at day 0 (for bovine, unconfined compression to 50% final strain at 100%/s strain rate; for human, 60% final strain at 300%/s strain rate; both followed by immediate release at the same rate [15,16]; treatment IC), and the two disease models receiving 100 ​nM Dex (treatments CD and ICD, respectively). Culture medium was collected every three or four days and stored at −20 ​°C until analysis.

### 2.3 Mass spectrometry preparation and identification

Culture medium (50 ​μL) was prepared for mass spectrometry (MS) analysis as described [3,16]. Discovery MS was performed on medium samples using a quadrupole Orbitrap benchtop mass spectrometer (Q-Exactive, Thermo Scientific). Identification was performed using the UniProt bovine (UP000009136, 2017–10) and human (UP000005640) sequence databases with Proteome Discoverer 2.2 (Thermo Scientific). The protein false discovery rate (FDR) was 0.01. Label-free protein abundance quantification was obtained by summing peak area intensities from all of the unique peptides for each protein.

### 2.4 Bioinformatics analysis

For both the bovine and human data sets, proteins were filtered out if they were exogenously added or not identified and quantified in at least 70% of samples, and missing values were imputed using the *k*-nearest neighbor method, with a *k* of 6 for the bovine data and 4 for the human data (Supplemental Methods) [17]. Protein abundance data were log_2_-transformed and principle component analysis (PCA) was performed on treatments N, C, and IC using the "prcomp" function [3]. Pairwise comparisons between treatments were performed on the summed peptide abundance over all timepoints. Statistical analysis was performed using the R package limma [18] and MATLAB (MathWorks). The amount of each protein released at each timepoint was normalized to the total amount released, then averaged across three biological replicates. Release vectors were clustered based on correlation using Euclidian distance (Fig. 1C). Cluster enrichment for proteins with increased or decreased release from control was determined by finding the number of proteins with a significant fold change from control in each cluster, selecting that number from the total population, and generating 10,000 bootstrapped distributions to determine the likelihood of having so many proteins with increased or decreased release [19]. Enrichment analysis was performed as previously described [3].

## 3 Results

### 3.1 Protein identification and differential analysis

**Fig. 2:**
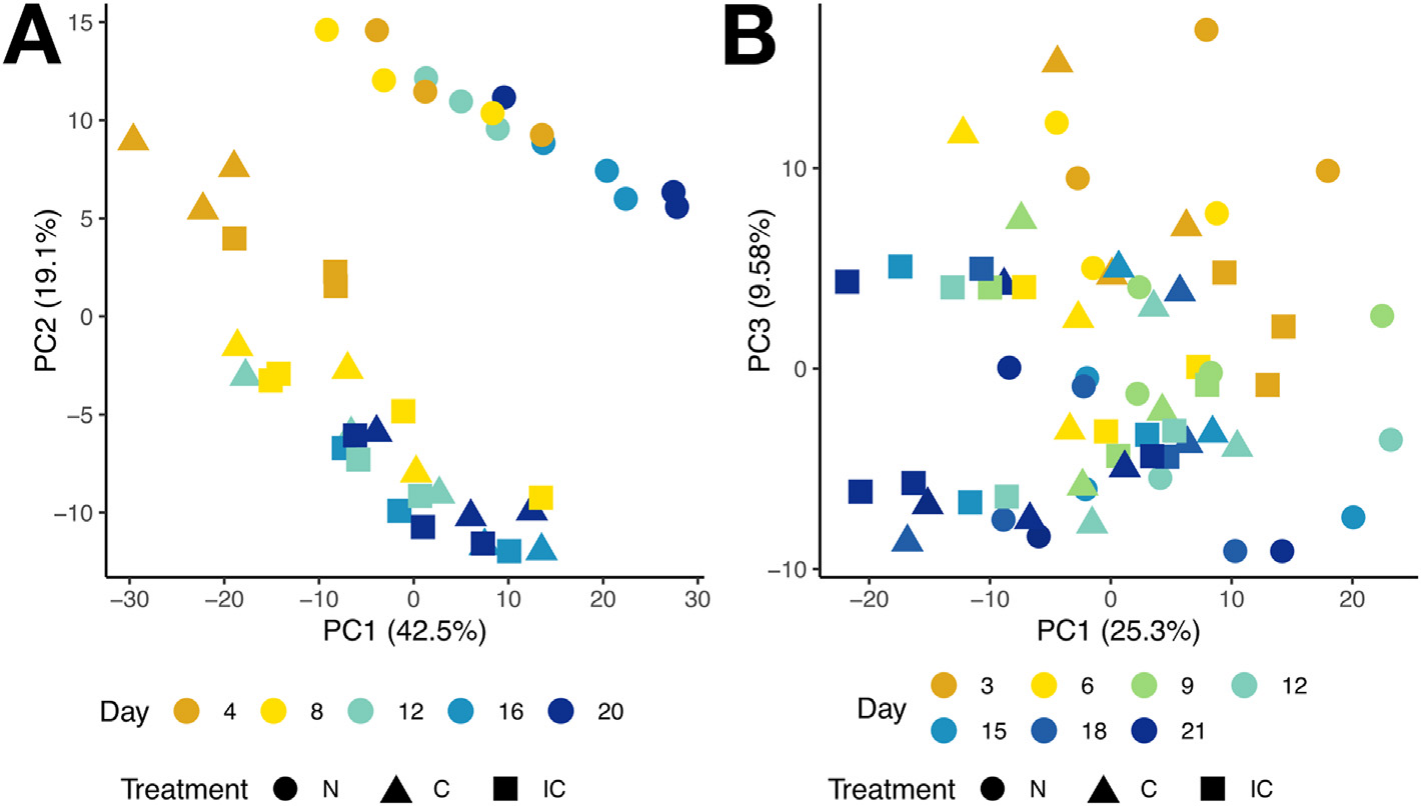
**Principal component analysis of proteomic data. (A)** PCA clustering was performed on bovine control (N), cytokine-treated (C), and injury ​+ ​cytokine-treated (IC) samples. C and IC samples separated from control samples, and there was a noticeable separation of early timepoints from later ones, particularly for treatments C and IC. **(B)** PCA on human N, C, and IC samples revealed a similar trend apparent when plotting the first and third principal components. Percentages on axes represent percent variance explained by that principal component.

Raw MS data are available via Proteome Xchange with identifiers PXD020756 and PXD024359. After filtering as described above, the data sets were reduced to 405 proteins for bovine samples and 416 proteins for human (Supplemental File S1). PCA clustering on log_2_-transformed control, C, and IC data for both bovine and human samples revealed a strong separation by time for bovine samples (Fig. 2A), with a less prominent trend plotting principal components 1 and 3 for human samples (Fig. 2B). Results from the differential analysis can be found in Supplemental File S2.

### 3.2 Bovine kinetic clustering

**Fig. 3:**
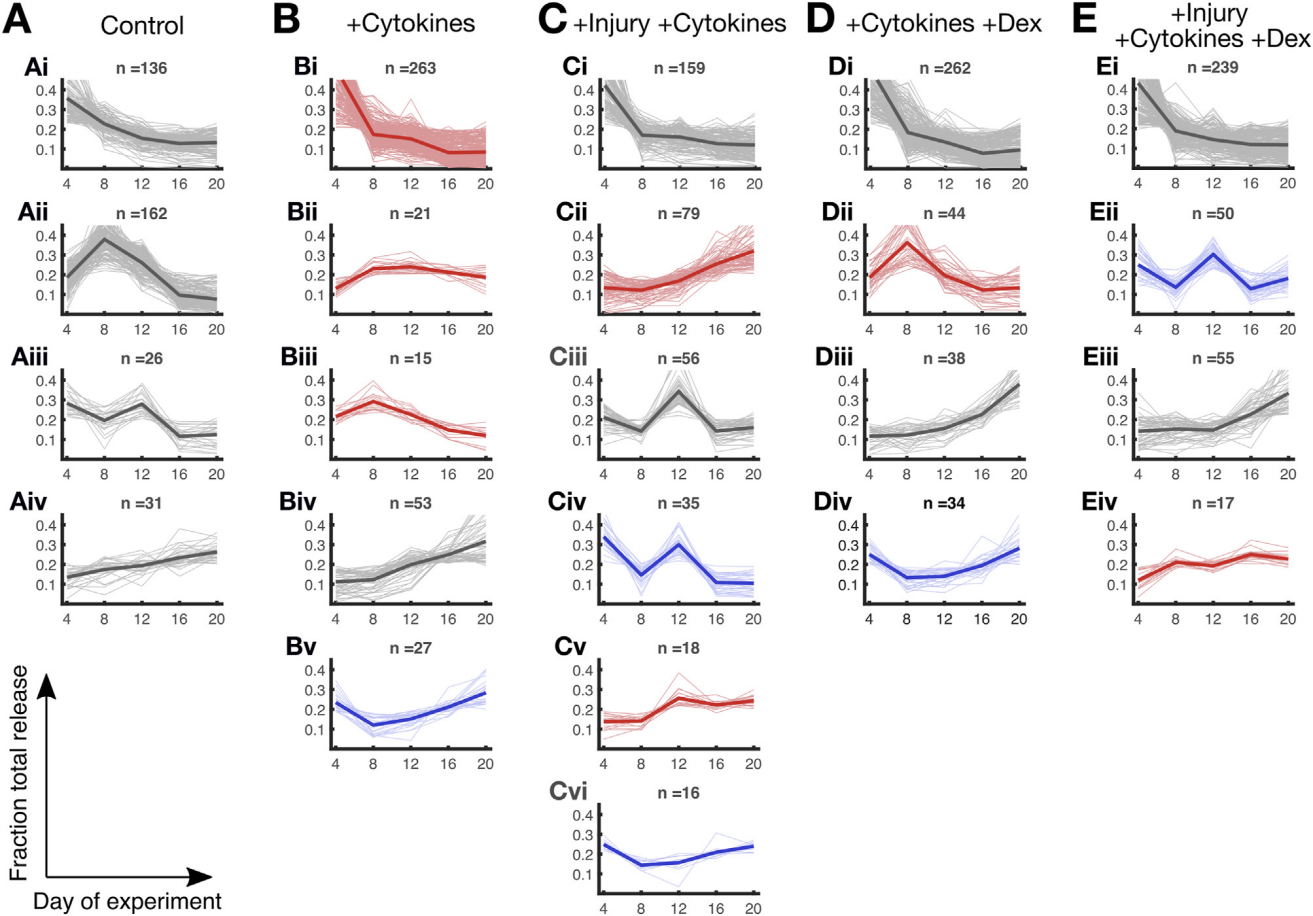
**Clusters of bovine protein time release profiles.** After clustering based on correlation, clusters of proteins with distinct time release profiles emerged for all treatment conditions: control **(A)**, cytokine-treated **(B)**, injury ​+ ​cytokines **(C)**, cytokines ​+ ​Dex **(D)**, injury ​+ ​cytokines ​+ ​Dex **(E)**. Cluster enrichment for proteins with increased (red) or decreased (blue) release from control was determined by finding the number of proteins with a significant fold change from control in each cluster and bootstrapping 10,000 random distributions based on the total protein population. Lighter lines represent each individual averaged vector, dark lines represent the average of all proteins in that cluster. Clusters are arranged in descending order based on timing of approximate peak release of proteins in that cluster and/or when a steadily increasing trend begins. X-axis: days of the experiment. Y-axis: fraction of total protein release collected each day. n: number of proteins in each cluster.

**Fig. 4:**
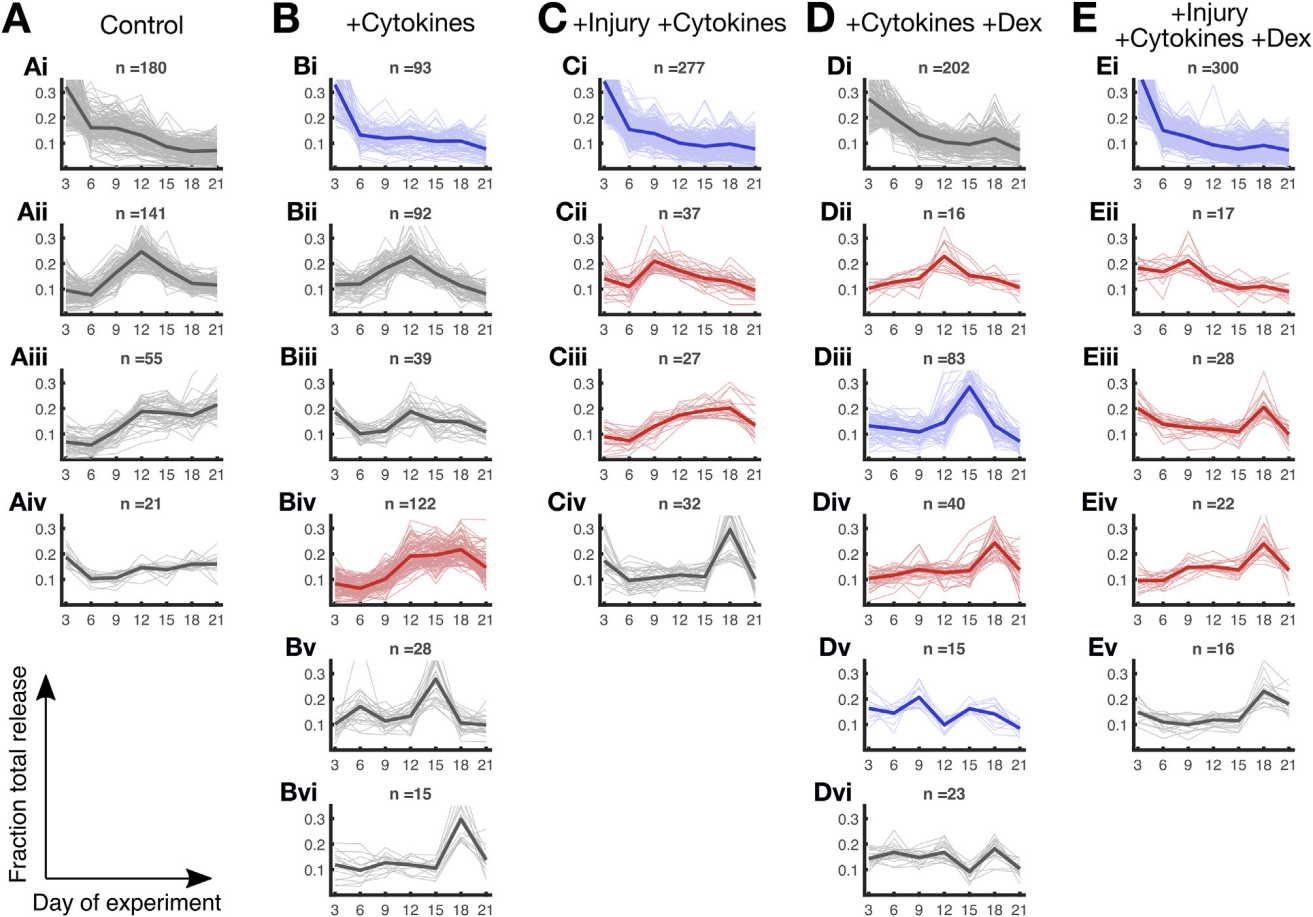
**Clusters of human protein time release profiles.** Time profile clustering of the human data: control **(A)**, cytokine-treated **(B)**, injury ​+ ​cytokines **(C)**, cytokines ​+ ​Dex **(D)**, injury ​+ ​cytokines ​+ ​Dex **(E)**. Red: cluster with significant number of proteins with increased release from control. Blue: cluster with significant number of proteins with decreased release from control. Lighter lines represent each individual averaged vector, dark lines represent the average of all proteins in that cluster. Clusters are arranged in descending order based on timing of approximate peak release of proteins in that cluster and/or when a steadily increasing trend begins. X-axis: days of the experiment. Y-axis: fraction of total protein release collected each day. n: number of proteins in each cluster.

To explore the time-dependent effects on protein release from bovine cartilage, we clustered the proteins based on the vectors of their averaged, normalized release at each timepoint (Fig. 1C). We included for discussion the clusters with fifteen or more members, as below that threshold few conclusions on enrichment of biological processes could be made (Fig. 3, Fig. 4, list of proteins in each cluster in Supplemental File S3). All clusters are visualized and listed in Supplemental Files S4 and S5. For the control condition, four major clusters emerged: a cluster with day 4 peak release that decreased steadily over time (Fig. 3Ai), clusters with peak release on day 8 (Fig. 3Aii) and day 12 (Fig. 3Aiii), and a cluster with a slight, steady increase in amount released (Fig. 3Aiv). Cluster **3Aii** was enriched for intracellular metabolic proteins.

Clusters for treatments C and IC are depicted in Fig. 3B and C. Clusters **3Bi**, **3Bii**, **3Biii**, **3Cii**, and **3Cv** were enriched for proteins with increased total release versus control, while **3Bv**, **3Civ**, and **3Cvi** had decreased release versus control. Many of the proteins in **3Ai** were found in clusters with increased release later in culture (**3Bii**, **3Biii**, **3Cii**), suggesting that these proteins are affected by disease processes that cause increased matrix breakdown and hence transport permeability. Clusters **3Biv** and **3Ciii** contain proteins without a significant change in total amount released including complement components C1R, C1S, B, and I, as well as transforming growth factor beta-2 for treatment C. Some ECM components with peak release on day 12 or an increase starting on day 12 (**3Bv** and **3Civ**) had decreased release versus control, including collagen II.

### 3.3 Dex effects on bovine release kinetics

The Dex-treated conditions (Fig. 3D, **E**) had large clusters of proteins that experienced peak release on day 4 (**3Di**, **3Ei**). These clusters contained many proteins that followed the diffusive pattern of release in control (**3Ai**), but experienced different release kinetics with C or IC treatment. Notable proteins that were not restored back to their diffusive behavior include collagens I and II and extracellular matrix protein 1 (ECM1), with steadily increasing release starting day 12 (**3Diii**, **3Eiii**). CD treatment yielded a cluster of proteins with increased total release from control peaking on day 8 (**3Aii**). A cluster enriched for similar proteins released after ICD treatment had increased release each day after day 12 (**3Eiii**), though the total amount released for these proteins was not significantly different than control. Many immune-related proteins, including complement factors C1R, C1S, C1Q, I, and pentraxin related protein PTX3, had late release (**3Diii**-**iv**, **3Eiv**). The total amount released for some proteins having CD treatment (**3Div**) was less than in control, but for some proteins with ICD treatment (**3Eiv**) was greater than in control.

### 3.4 Validation with human data set

After observing that this clustering method could distinguish different profiles of release kinetics in our bovine proteomic data set, we applied the same methods to the human medium proteome to determine whether this method would reveal proteins having shared release kinetics, and whether those human clusters would be similar to bovine. Four distinct protein clusters were found in the control condition (Fig. 4A), the largest with peak release on day 3 (**4Ai**), a cluster with peak release on day 12 (**4Aii**), one with increasing release starting on day 6 (**4Aiii**), and the last with relatively constant release across all timepoints (**4Aiv**). Cluster **4Ai** (with early peak release) was similar to bovine cluster **3Ai**, containing ECM components and signaling factors. The human day 12 peak cluster (**4Aii**) shared similarities to the bovine day 8 peak cluster (**3Aii**), enriched for many of the same intracellular proteins. For both C and IC treatment conditions (**4B** and **4C**, respectively) large clusters were present with peak release at day 3, enriched for matrix proteins exhibiting decreased release versus control (e.g., collagens VI, IX, and XI). The cytokine-treated samples had two clusters with intracellular proteins peaking on day 12 (**4Bii-iii**) not present with the addition of mechanical injury. The increased release of proteases and matrix proteins, including aggrecan, metalloproteinases, and collagen I (Supplemental File S2), was apparent in a cluster with a peak at day 12–18 (**4Biv**), and in two clusters that had an earlier release for injury ​+ ​cytokines (**4Cii-iii**). Both models of disease had late release of cathepsins and immune proteins peaking at day 18 (**4Bv-vi** and **4Civ**), but with no change in total release of these proteins versus control.

### 3.5 Dex effects on human release kinetics

Similar to results with bovine explants, many of the proteins in cluster **4Ai** experienced a change in release kinetics with C or IC treatment that was attenuated with addition of Dex (**4Di, 4Ei**), with the notable exceptions of several proteases, some collagens, and immune factors (MMPs −2, −3, −10, collagens III, IX, XII, serum amyloid A, beta-2-microglobulin, complement factors 3, C1S, C1R). Both Dex treatment regimens caused a group of proteins including MMP-2 and -3 to experience peak release on day 12 or day 8 (**4Dii**, **4Eii**), that had increased release versus control. These proteins had decreased total release compared to their non-Dex treated counterparts. The Dex-treated conditions also had clusters with peak release on day 18 with many immune proteins, proteases, and protease inhibitors with increased release versus control (**4Div**, **4Eiii-v**). With treatment CD, some proteins had relatively steady release across the entire culture (**4Dv**-**vi**).

### 3.6 Release kinetics of selected spatially and functionally related proteins

**Fig. 5:**
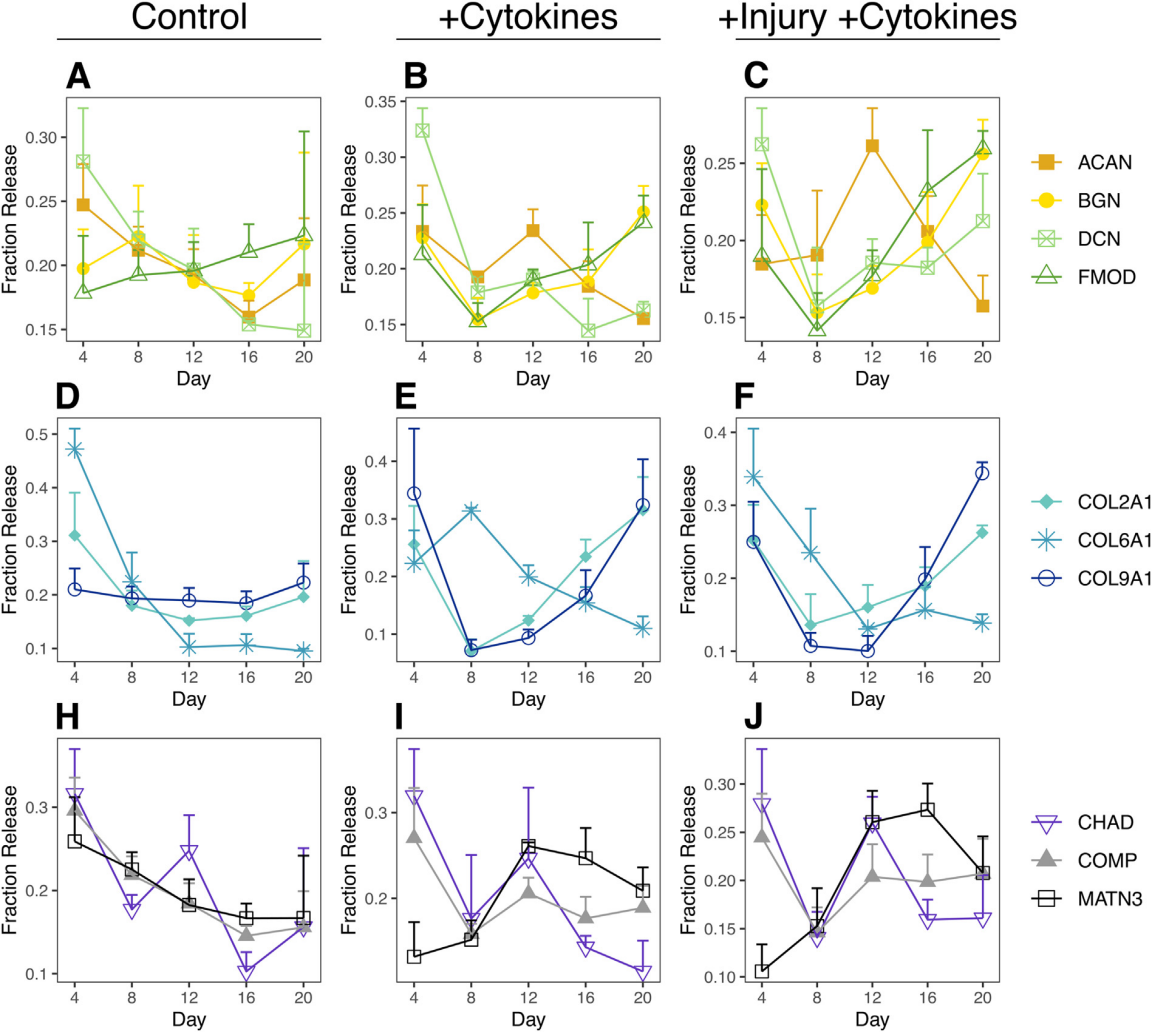
**Kinetics of selected proteoglycans, collagens, and matrix-binding proteins from bovine model.** Averaged normalized protein release vectors for aggrecan (ACAN), biglycan (BGN), decorin (DCN), fibromodulin (FMOD), collagens II, VI, and IX (COL2A1, COL6A1, COL9A1), chondroadherin (CHAD), COMP, and matrilin-3 (MATN3). **A-C:** proteoglycans. **D-F:** collagens. **H-J:** matrix-binding proteins. Columns represent untreated control (**A, D, H**), cytokine (**B, E, I**), and injury ​+ ​cytokine treatments (**C, F, J**). Error bars: standard deviation across three replicates.

**Fig. 6:**
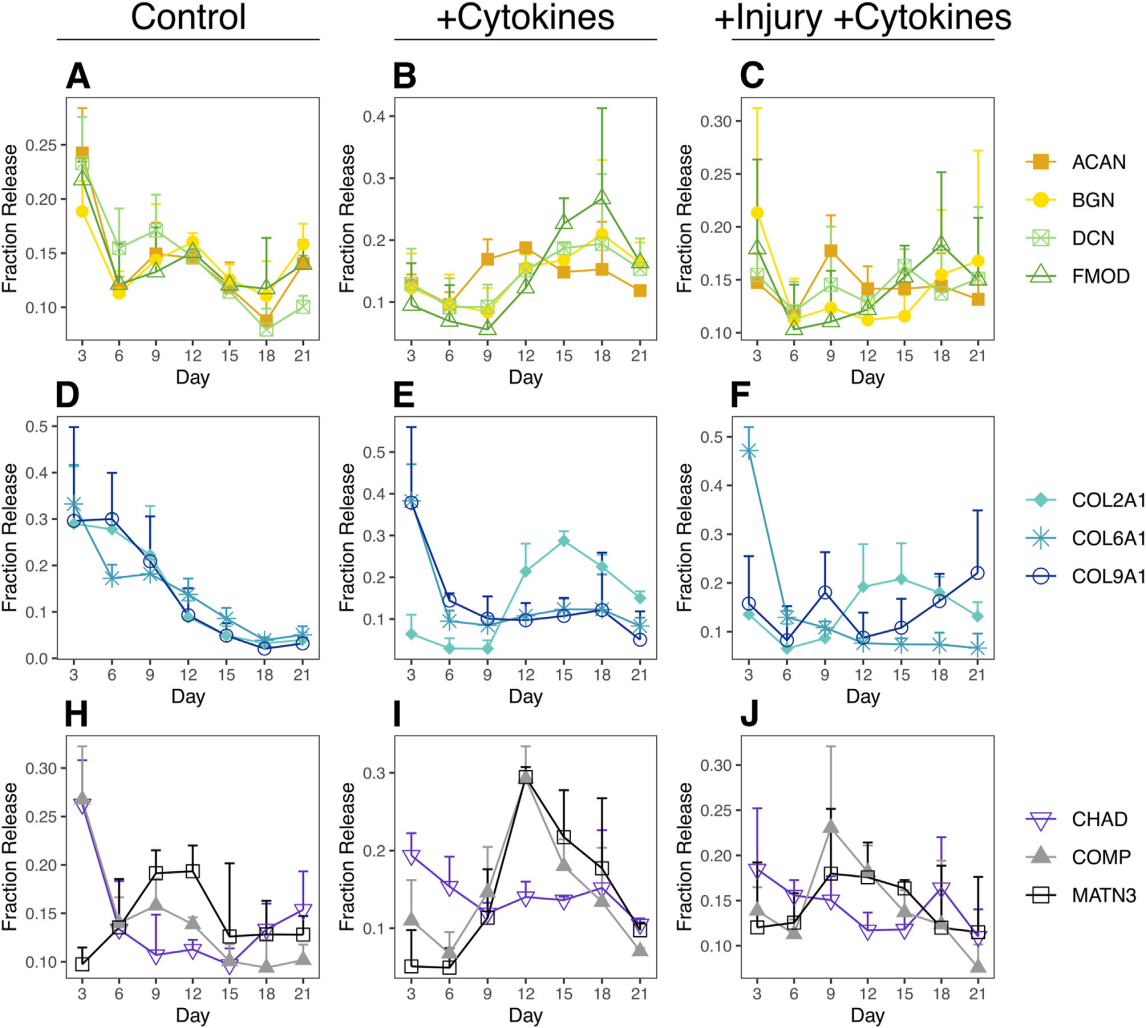
**Kinetics of selected proteoglycans, collagens, and matrix-binding proteins from human model.** Averaged normalized protein release vectors for aggrecan (ACAN), biglycan (BGN), decorin (DCN), fibromodulin (FMOD), collagens II, VI, and IX (COL2A1, COL6A1, COL9A1), chondroadherin (CHAD), COMP, and matrilin-3 (MATN3). **A-C:** proteoglycans. **D-F:** collagens. **H-J:** matrix-binding proteins. Columns represent untreated control (**A, D, H**), cytokine (**B, E, I**), and injury ​+ ​cytokine treatments (**C, F, J**). Error bars: standard deviation across three replicates.

Heinegård [20] reviewed macromolecular constituents of the cartilage extracellular matrix; to investigate when spatially and functionally related matrix components were released from cartilage explants, we examined the kinetics of release of selected proteoglycans, collagens, and matrix-binding proteins that are well-characterized. Selected were the large proteoglycan aggrecan (ACAN), and the small leucine-rich repeat (SLRR) collagen-binding proteoglycans biglycan (BGN), decorin (DCN), and fibromodulin (FMOD). Collagens selected were collagen II (COL2A1), collagen VI (COL6A1), and collagen IX (COL9A1). Additional matrix-binding proteins were the collagen-binding proteins COMP and matrilin 3 (MATN3), and the integrin-binding chondroadherin (CHAD). Proteins were selected if they were present in both bovine and human systems to enable comparison: Fig. 5 (bovine) and Fig. 6 (human).

Overall, the selected proteoglycans released from bovine cartilage with cytokine or injury ​+ ​cytokine treatment behaved similarly to control (Fig. 5A–C). Aggrecan had a peak in the amount released on day 12 in the diseased treatments, with a moderate amount released on days 4 and 8. Particularly in the injury ​+ ​cytokine treated samples (Fig. 5C), the SLRR proteoglycans had a large amount released at the earliest timepoint but a steadily increasing rate of release later in culture after the peak in aggrecan release, which was also reflected in the behavior of collagens II, VI, and IX (Fig. 5E–F). In untreated bovine controls, the collagens except collagen IX followed a diffusion-like pattern of release (Fig. 5D). The additional matrix binding proteins (Fig. 5H–J) behaved similarly to control with cytokines and/or mechanical injury, except with relatively more matrilin-3 released at later timepoints than in control (~25% of total release occurring at each of days 12 and 16).

In untreated human control explants (Fig. 6A, D, H), nearly all proteins had their peak release at the first timepoint (day 3). This behavior changed noticeably with inflammatory cytokines and mechanical injury: collagen VI, IX, and CHAD still experienced peak release at day 3, but other proteins had a peak release at days 12–18. With cytokine-only treatment, aggrecan release peaked on day 12, similarly to the peaks of COMP and matrilin-3 on day 12. These peaks were followed by a day 15 peak in collagen II release and later increases in decorin, biglycan, and fibromodulin release (Fig. 6B, E, I); this trend was less noticeable with mechanical injury (Fig. 6C, F, J).

Overall, the addition of Dex did not have a major impact on the release of most of these selected proteins in the bovine model (Figs. 5, **6**, and Supplemental File 7). In the human model, Dex changed the behavior of nearly all selected proteins to have peak release at the earliest timepoint, with some proteins (e.g. CHAD) experiencing a small increase at day 18.

## 4 Discussion

Our clustering method allowed us to analyze existing bovine and human proteomic data sets for information on matrix breakdown kinetics and potential biomarker candidates, which could easily be adapted to new data sets. The data from the explant model of human PTOA closely match the kinetics of matrix breakdown for *in vivo* knee injury, as measured by COMP, osteopontin, osteonectin, complement factor, and collagenase-generated collagen II fragment release into the synovial fluid [[21], [22], [23], [24], [25]] [[21], [22], [23], [24], [25]] [[21], [22], [23], [24], [25]]. Thus, our results from the human model closely follow the same breakdown events that occur in patients, though the timing is likely not 1:1 as the presence of other joint tissues could affect the rate of catabolic processes.

In general, the human and bovine results had the same patterns of release. Both sets of control data showed a large cluster of proteins with peak release at the earliest timepoint. Proteins with this profile are assumed to have no direct biological or biochemical initiators of loss from explant culture, most likely following passive diffusion-like transport out of the cartilage. This diffusive profile may be associated in part with the newly-cut radial edges of the cartilage plugs upon harvest. Interestingly, this profile may also model initial diffusive loss *in vivo* when traumatic knee injury results in cracks in the cartilage surface, as can commonly occur [1,26]. In addition, both human and bovine untreated conditions also had a group of intracellular proteins of low abundance that had peak release about one week into the culture, assumed to be indicative of early cell death [3].

The bovine and human C and IC conditions had large clusters of proteins peaking least one week into culture. The human clusters often had sudden, sharp increases in the amount of protein released, while the bovine proteins had more gradual increase over time. The proteins in these clusters are the most likely candidates for biomarkers of PTOA progression due to the increased amount released compared to control. Notably, the addition of a single mechanical impact injury caused matrix breakdown to begin several days sooner than cytokines alone. This may be associated with injury-induced microdamage to the matrix, enabling enhanced transport of cytokines into cartilage and release of matrix breakdown products, or an increase in intracellular catabolic processes with mechanical stimulus. Treatment with cytokines alone may also model OA progression influenced by inflammation due to the shared pathways that will be affected by exposure to inflammation, though it is difficult to apply our analysis of the kinetics of release of biomarkers to this disease model, as the timing of disease initiation is not as distinct as with PTOA.

This method of biomarker identification is validated by the overlap of our results with previously posited ECM-related biomarker candidates such as aggrecan, MMP-3, collagen II, vascular cell adhesion protein-1, lumican, and COMP [[27], [28], [29], [30]] [[27], [28], [29], [30]] [[27], [28], [29], [30]]. Other biomarker candidates identified by our method are cytokines that may be part of the early breakdown signaling process, such as IL-8, secreted phosphoprotein-1, PTX3, and C–C motif chemokine ligands 5 and 20 [[31], [32], [33], [34], [35]]. Our study suggested the importance of biomarkers related to cell death as well as matrix breakdown, such as cathepsins L1, D, and S, which may be indicators of cellular response to PTOA progression and are under consideration as potential non-invasive biomarkers for early OA [36,37]. A novel set of candidate markers of PTOA progression identified with this method as well as previous proteomic analysis of the bovine data set [3] were lamin and histone proteins present in the culture medium (LMNA, LMB2, HIST1H4A, HIST1H1D, H2AFV, H3FA, HIST2H2BF). The presence of these nuclear proteins in culture medium could be indicative of cell death in the early stages of cartilage damage, and they have been identified in previous proteomic studies of synovial fluid in OA models [[38], [39], [40]] [[38], [39], [40]] [[38], [39], [40]].

Just as critical as the biomarker identity itself is the timing of its release into synovial fluid. Based on our human data, increased levels of matrix degradation products and signaling proteins that have been previously identified as biomarker candidates peaked in their release 9–15 days after the initial injury. While some biomarkers have been identified in synovial fluid months to years after injury [8], it is difficult to maintain an *ex vivo* model this long, limiting our window of investigation to relatively early timepoints. However, there is reason to investigate this window of time for identifying patients with an elevated risk for PTOA development, as early intervention with a drug such as Dex might prevent irreversible damage to the cartilage that could occur.

Some matrix components such as collagen II in the bovine C and IC conditions experienced a decrease in their total amount released compared to control. As previously reported [3], some ECM components experience a decreased release under inflammatory and mechanical stress due to a decrease in synthesis. However, the kinetics of the release of collagen II did change (Figure 3Bv, **3Cvi**, **6E**-**F**), demonstrating that collagen II release was still affected by protease activity and disease progression. Particularly in the bovine model, where collagen II synthesis is high, the release of collagen II into the matrix will be dependent on its synthesis as well as its proteolytic cleavage and matrix permeability. Within the selected matrix proteins, some proteins that decorate the surface of larger ECM macromolecular complexes (e.g., COMP, collagen IX, and matrilin-3) experienced their peak release before collagen II (Figs. 5 and 6E, F, I, J) [41]. The sequential loss of specific matrix components supports the hypothesis that highly abundant and space-filling matrix proteins such as aggrecan must first be degraded and released to some extent before proteases can then reach the cleavage sites of other proteins such as collagen II, decorin, biglycan, and fibromodulin [42].

The addition of a low dose of Dex rescued the increased release of many matrix breakdown products, though not entirely to control levels, in both the human and bovine model. This is in agreement with previous studies showing that Dex reduced the activity of matrix proteases and attenuating collagen and GAG loss in models of OA/PTOA [[2], [3], [4], [10], [16]] [[2], [3], [4], [10], [16]] [4,10,16]. Further evidence that Dex rescues ECM homeostasis to control is demonstrated by many proteins with their kinetics of release affected by cytokines and/or injury returning to their control behavior (Fig. 3Di, **3Ei**, **4Di**, **4Ei**), though some MMPs and collagens still had release later than they did without treatment.

The most significant off-target effect of Dex we observed was the increased release of inflammatory mediators late into culture in the human model, where both CD and ICD conditions had clusters enriched for inflammatory proteins with a day 18 peak release. Dex has been shown to have anti-inflammatory effects on the joint space in animal models of PTOA [43], but the specific pathways through which it exerts this effect are currently not well-understood [2], and likely dependent on many tissues including the joint capsule synovium. This deviation from the signaling and release of inflammatory proteins in healthy, untreated cartilage justifies more studies on appropriate doses and timing of Dex and the interaction of Dex with multiple joint tissue types [44]. If Dex is to be used for its anti-catabolic effects to protect against PTOA progression and avoid potential off-target effects on immune signaling or other pathways, targeted strategies of drug delivery specifically to cartilage at the lowest possible dose will be necessary [[45], [46], [47]]. ‬‬‬‬‬‬‬‬‬‬‬‬‬‬‬‬‬‬‬‬

### 4.1 Study limitations

Mass spectrometry as a technique has specific limitations. We used a high threshold of confidence to compensate for any single-peptide identifications or missing values, with a false discovery rate of 0.01 and proteins required to have quantification values in at least 70% of samples. Some putative OA biomarkers are specific cleavage fragments of matrix components, such as CTX-II [29] and COMP neoepitopes [16,23]; with our broad discovery approach, these peptides would not be distinguished from the rest of the peptides used to calculate protein abundance due to trypsin cleavage. Thus, this method for biomarker identification is best validated with other biochemical or targeted proteomic analyses [3]. The bovine experiment used three biological replicates, a low number for statistical confidence but balanced by the repeatable nature of these juvenile bovine experiments, and our human data set used only one donor with three internal technical replicates. Donor-to-donor variability can be extremely high, and our ongoing studies with more donor joints will be used to further validate our application of the clustering method used here. The difference in cytokine concentrations and culture conditions between the models was due to the difference in young juvenile chondrocytes versus aged and potentially more sensitive adult human cells. These differences in age, developmental state and phenotype will contribute to differences between our models, such as differences in baseline synthesis rates of collagen II between less metabolically active adult human cartilage and the young bovine system, which may be more similar to human children or adolescents [48]. Hypoxic conditions in the *in vivo* joint environment may affect some of the pathways discussed. Ongoing studies including bone and synovial tissue will elucidate the effect of crosstalk from these tissues on cartilage response and the media proteome and further refine this model of PTOA progression [44].

## 5 Conclusions

Our analysis identified the presence of distinct clusters of proteins based on the kinetics of their release over three weeks of culture. In our model of early PTOA progression, our data suggest that many biomarkers for cartilage matrix breakdown are released at their peak nine to fifteen days after initial injury. Utilizing this method allows for broad identification of biomarker candidates for early monitoring of PTOA risk in patients after a traumatic joint injury. We found that a period between 9 and 15 days post-injury had the highest relative release of previously identified and novel biomarker candidates in our model. Our study also provided insight into the sequential loss of matrix constituents, where some proteins such as collagen II are shielded from breakdown and release into the media until other matrix components are degraded. Dex showed promise as an anti-catabolic disease-modifying treatment, restoring most ECM and ECM-modifying proteins to their control behavior and amount released. However, in all treatment conditions except control, there was a late peak in the release of complement factors, suggesting an immune response in cartilage that is not attenuated with Dex treatment. This highlights the need to better understand the effect of Dex on other pathways involved in cartilage homeostasis beyond matrix breakdown, and the potential need to target these pathways with additional combinatorial therapeutics [49]. The clustering results had the same broad trends between human and bovine models, but the release kinetics for specific proteins often differed; a bovine model may not be appropriate to search for OA biomarkers without validation against human data.

## 6 Contributions

All were involved in the conception and design of the study as well as interpretation of the data. YW, PL, AS conducted the experimental work and data analysis. PÖ conducted the mass spectrometry work and data analysis. Bioinformatics analysis was performed by RMB. RMB drafted the manuscript while all authors critically revised the manuscript and gave final approval of the article.

## Declaration of Competing interest

The authors do not have any conflict of interest.
