# Supplementary material for: Proteomic clustering reveals the kinetics of disease biomarkers in bovine and human models of post-traumatic osteoarthritis
Source: Osteoarthr Cartil Open. 2021 Jun 10;3(4):100191. doi: 10.1016/j.ocarto.2021.100191 (PMC9611763; doi:10.1016/j.ocarto.2021.100191)
Supplement: Multimedia component 1 [file mmc1.docx]

**Supplemental Methods**: Media composition

After harvesting, explant disks were pre-equilibrated in serum-free phenol-red free Dulbecco’s Modified Eagle’s Medium (DMEM, 1 g/L glucose for bovine and 4.5 g/L glucose for human, ThermoFisher Scientific) supplemented with 10 mM HEPES buffer (Gibco), 0.1 mM nonessential amino acids (Sigma), 0.4 mM proline (Sigma), 20 µg/ml ascorbic acid (Sigma), 100 units/ml penicillin G, 100 µg/ml streptomycin, and 0.25 µg/ml amphotericin B (Sigma), and 1% insulin-transferrin-selenium (10 µg/ml, 5.5 µg/ml, and 5 ng/ml, respectively; Sigma)[3,13]. In addition, bovine media received 2 mM L-Glutamine (Gibco) and human media received 1 mM sodium pyruvate (Gibco). After the equilibration period, bovine explants were cultured with low-glucose (1 g/L) DMEM and human maintained in high glucose medium, based on precious controls comparing high and low glucose treatment with human explants[4].

**Supplemental Methods**: imputation via *k*-means clustering

*k* values for *k*-means clustering of the proteomic data were obtained via the testing method described in Lazar et. al, 2016[15]. The proteomic data for both bovine and human data were filtered to only include proteins with no missing values, and then missing values were generated based on the total number of missing values in the original data set after filtering out proteins with more than 30% missing values. Different proportions of missing values were generated at random versus not at random (40%, 50%, 60%, 70%, 80%, 90% of missing values not at random), with the not-at-random threshold set by the lowest abundance remaining in the data value if all missing values were assigned to the values with the lowest abundance data.

After generating a data set with different proportions of random versus not at random missing values, the data was log2 transformed, normalized, and *k* values between 1 and 16 were used to impute the missing values. The root mean square error between the original data and imputed data was then calculated. *k* values of 6 for bovine and 4 for human were selected based on their consistent lowest RMSE at any given missing not at random fraction.

Both data sets were collected originally with an injury alone treatment condition with or without Dex, but these were excluded from our analysis due to previous work finding a relatively small impact of injury alone in this PTOA model[3]. After removing those treatment conditions, proteins were filtered out if they were exogenously added or not identified and quantified in at least 70% of samples, then imputation was performed.
