# Supplementary material for: Proteomic clustering reveals the kinetics of disease biomarkers in bovine and human models of post-traumatic osteoarthritis
Source: Osteoarthr Cartil Open. 2021 Jun 10;3(4):100191. doi: 10.1016/j.ocarto.2021.100191 (PMC9611763; doi:10.1016/j.ocarto.2021.100191)
Supplement: Multimedia component 2 [file mmc2.docx]

**Supplemental File Legends**

**File S1**: Imputed proteomic data sets for bovine (405 proteins) and human (416) experiments. N: no treatment; C: cytokine treatment; IC: injury + cytokine treatment; CD: cytokine+Dex treatment; ICD: injury + cytokine + Dex treatment.

**File S2**: TopTable statistical summary of pairwise treatment comparisons. Log_2_-fold changes are reported for each pairwise comparison, as well as average log_2_-transformed release across all conditions, F statistic, raw and adjusted *p*-values, and log-odds value (‘B’). N: no treatment; C: cytokine treatment; IC: injury + cytokine treatment; CD: cytokine+Dex treatment; ICD: injury + cytokine + Dex treatment.

**File S3**: List of proteins in each cluster as represented in **Figures 3** and **4**. Clusters with 15 or more proteins were selected and ordered based on the timing of the approximate peak release of each cluster. C: cytokine treatment; IC: injury + cytokine treatment; CD: cytokine+Dex treatment; ICD: injury + cytokine + Dex treatment.

**File S4**: List of proteins in each cluster for each treatment condition, ordered by number of proteins in each cluster. N: no treatment; C: cytokine treatment; IC: injury + cytokine treatment; CD: cytokine+Dex treatment; ICD: injury + cytokine + Dex treatment.

**Fille S5**: Clusters for all proteins for bovine and human treatment conditions. Lighter lines represent each individual averaged vector, dark lines represent the average of all proteins in that cluster. X-axis: days of the experiment. Y-axis: fraction of total protein release collected each day. N: no treatment; C: cytokine treatment; IC: injury + cytokine treatment; CD: cytokine+Dex treatment; ICD: injury + cytokine + Dex treatment.

**Fille S6**: Averaged normalized protein release vectors for aggrecan (ACAN), biglycan (BGN), chondroadherin (CHAD), collagen II (COL2A1), collagen VI (represented by COL6A1), collagen IX (represented by COL9A1), cartilage oligomeric matrix protein (COMP), decorin (DCN), fibromodulin (FMOD), and matrilin -3 (MATN3) across all treatment conditions for bovine (**A-J**) and human (**K-T**) samples, organized by protein. N: control, C: cytokine treatment, IC: injury + cytokine treatment, CD: cytokine + Dex treatment, ICD: injury + cytokine + Dex treatment. Error bars: standard deviation across three replicates.

**Fille S7**: Averaged normalized protein release vectors for aggrecan (ACAN), biglycan (BGN), decorin (DCN), fibromodulin (FMOD), collagens II, VI, and IX (COL2A1, COL6A1, COL9A1), chondroadherin (CHAD), cartilage oligomeric matrix protein (COMP), and matrilin-3 (MATN3). **A-D:** proteoglycans. **E-H:** collagens. **I-L:** matrix-binding proteins. Columns represent cytokine+Dex (**A, E, I** and **C, G, K**) and injury+cytokine+Dex treatments (**B, F, J** and **D, H, L**). Error bars: standard deviation across three replicates. Error bars: standard deviation across three replicates.
