# Supplementary material for: Proteomic clustering reveals the kinetics of disease biomarkers in bovine and human models of post-traumatic osteoarthritis
Source: Osteoarthr Cartil Open. 2021 Jun 10;3(4):100191. doi: 10.1016/j.ocarto.2021.100191 (PMC9611763; doi:10.1016/j.ocarto.2021.100191)

# Bovine, Treatment **N**

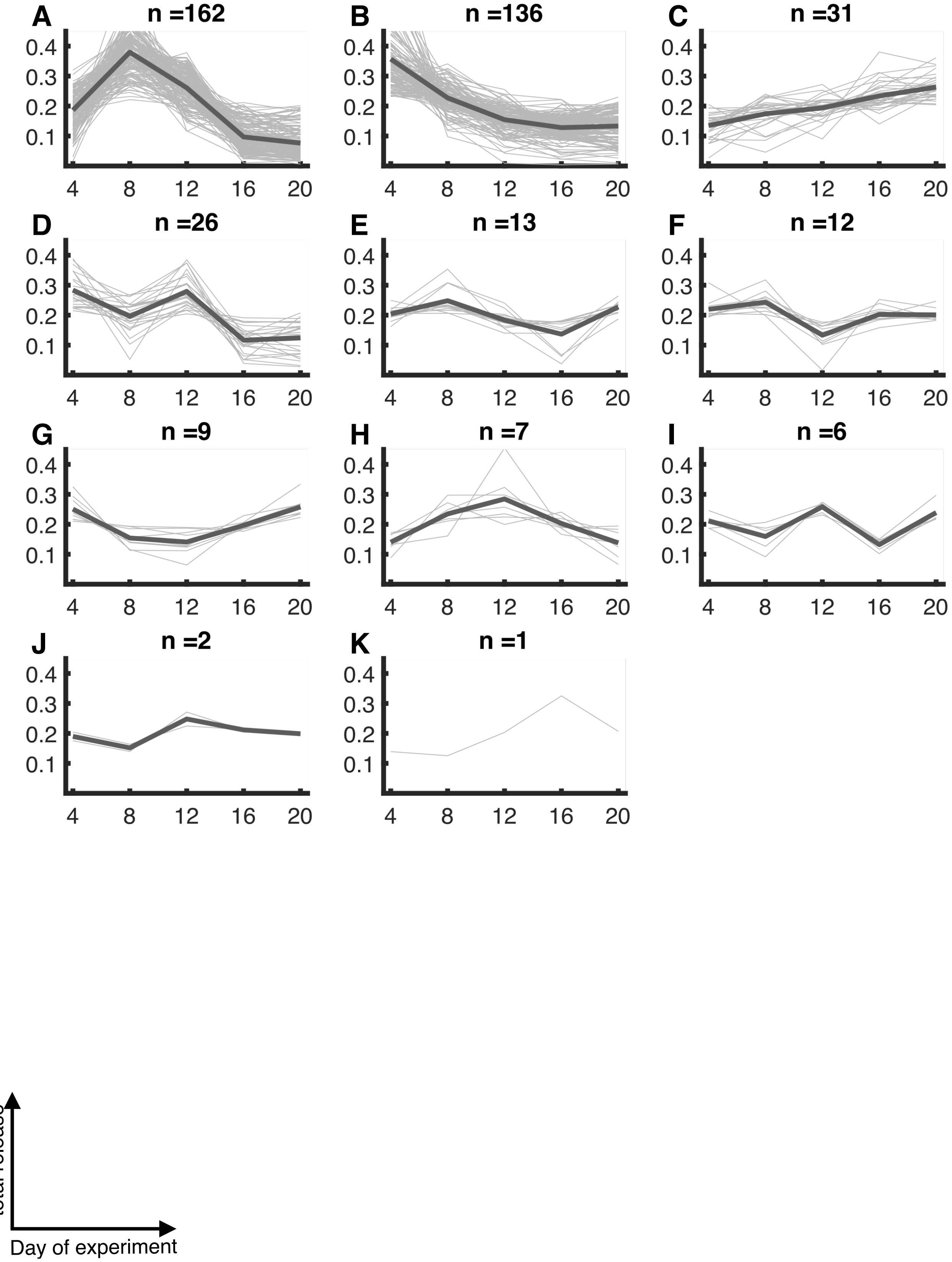

# Bovine, Treatment **C**

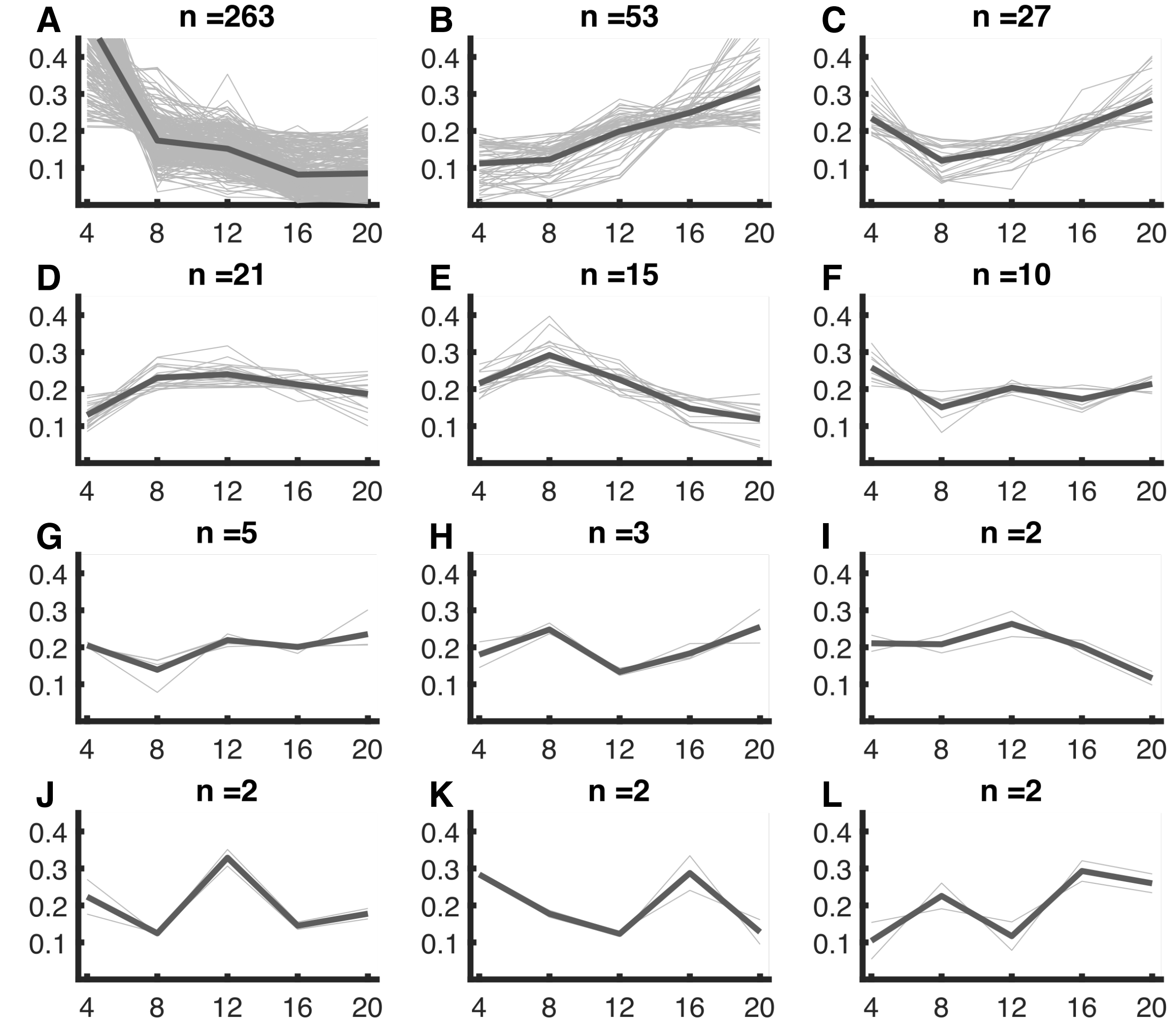


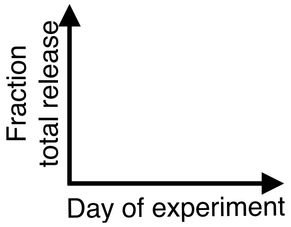

# Bovine, Treatment **IC**

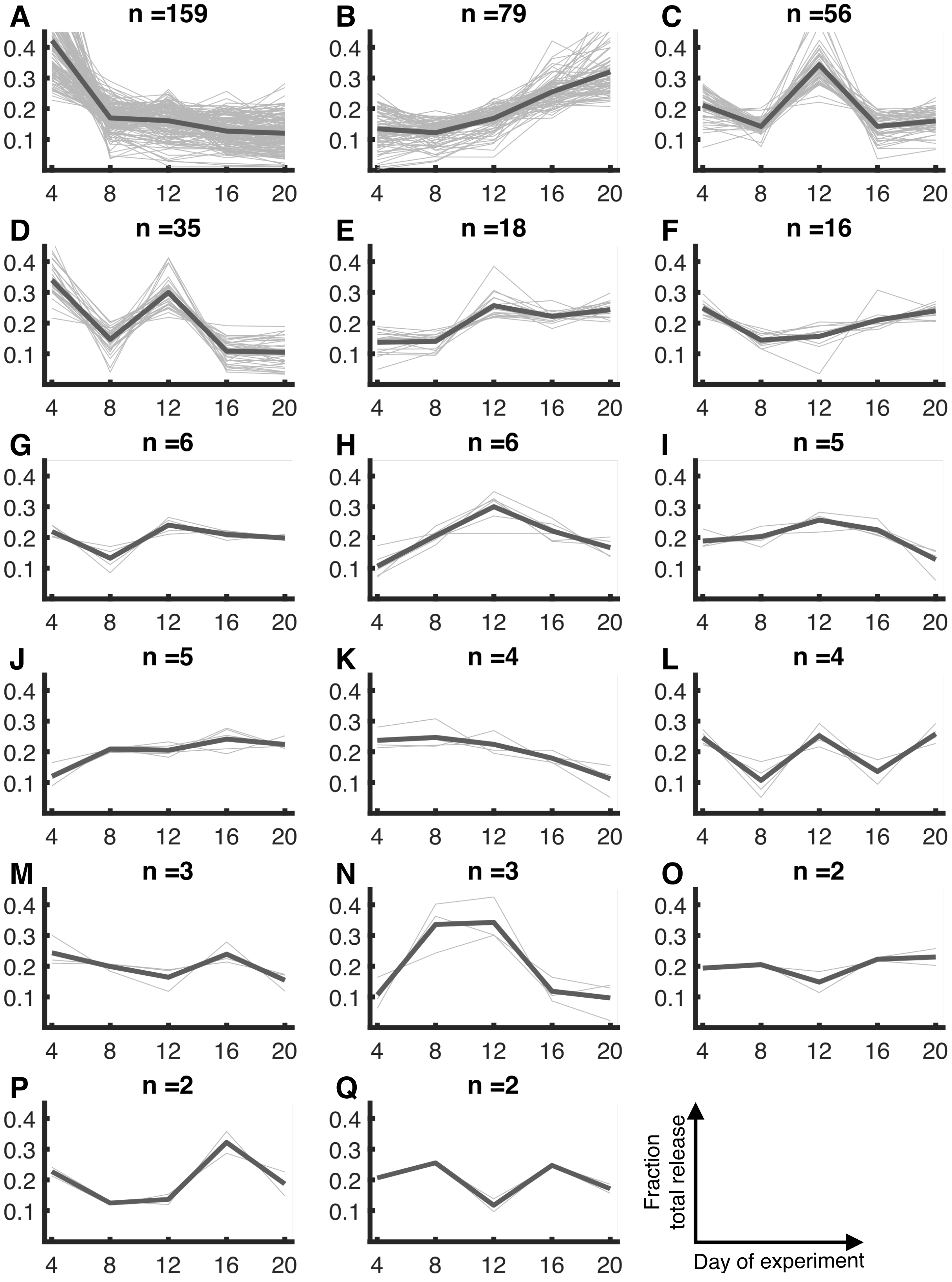

# Bovine, Treatment **CD**

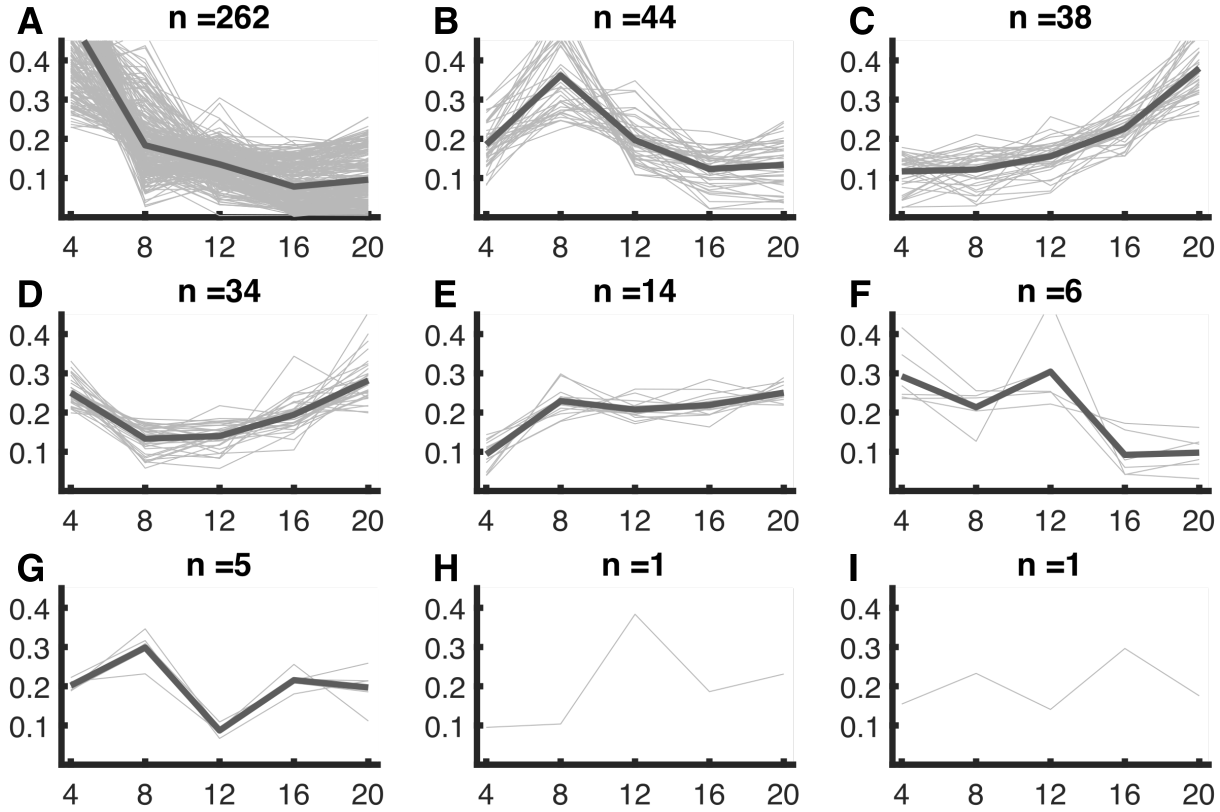


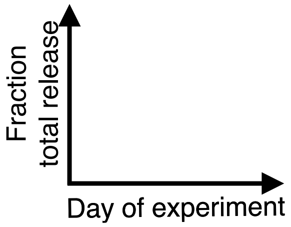

# Bovine, Treatment **ICD**

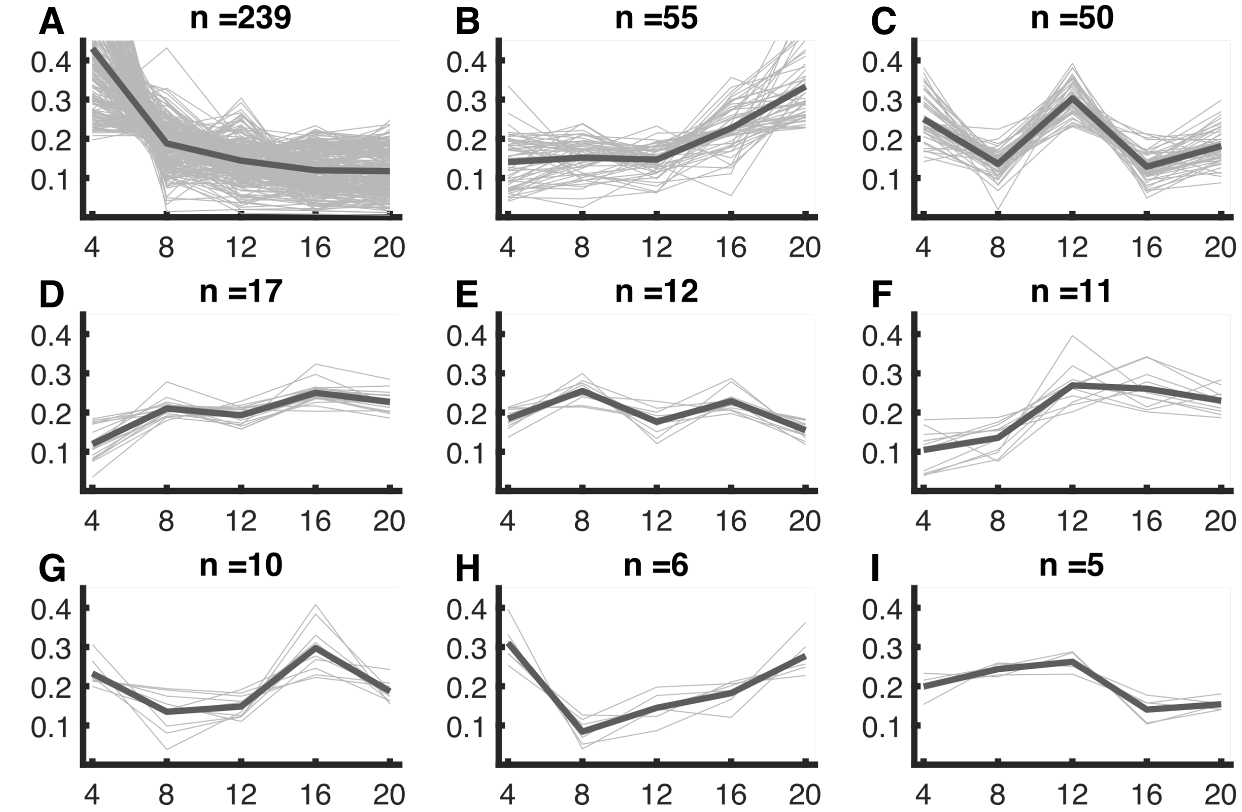


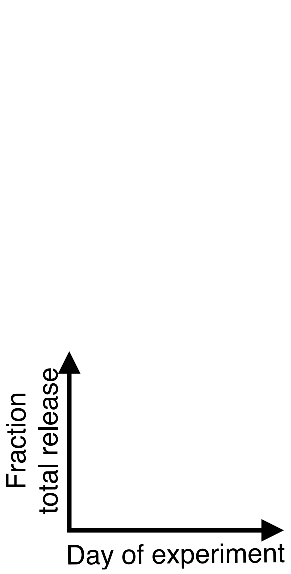

# Human, Treatment **N**

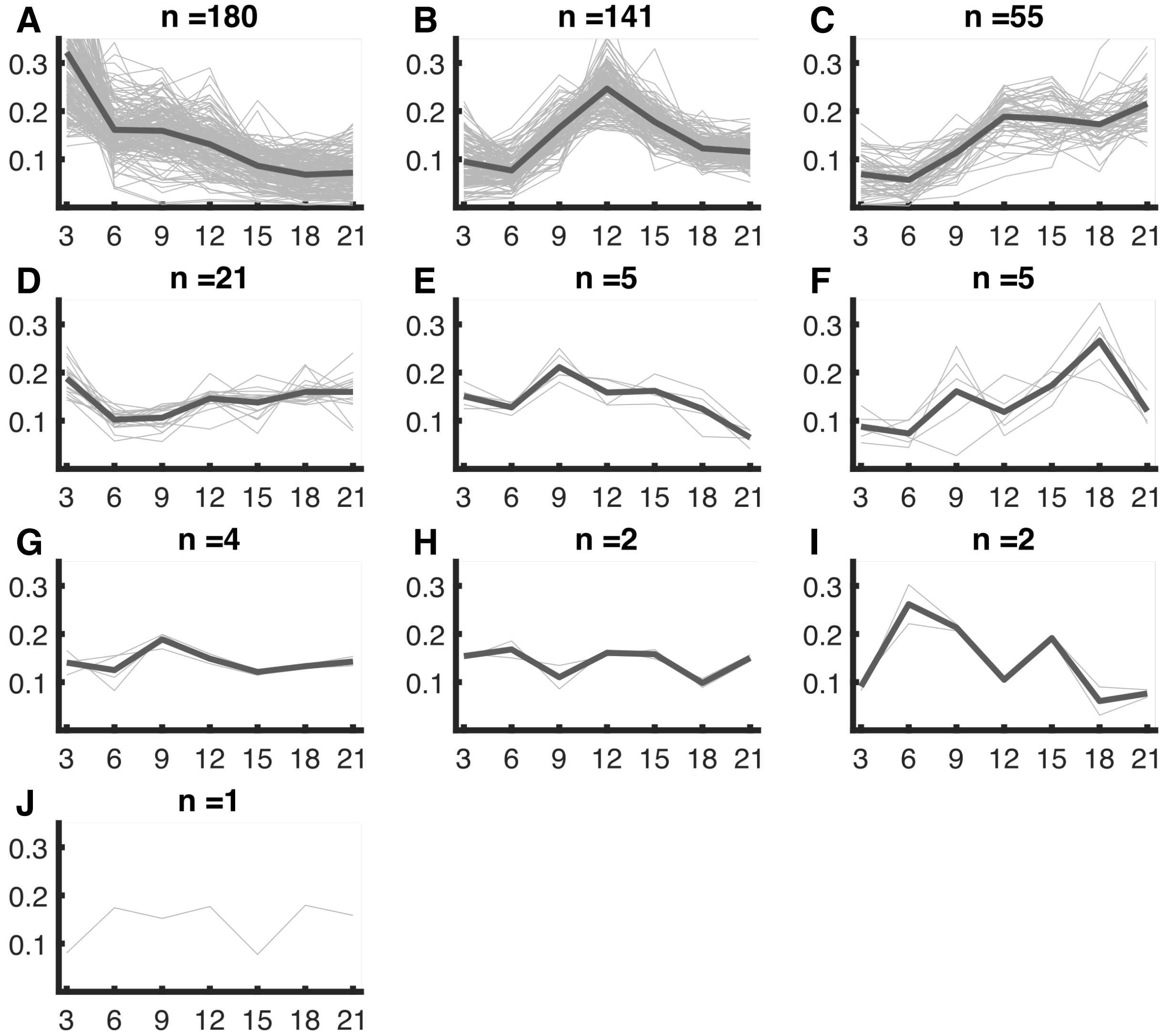


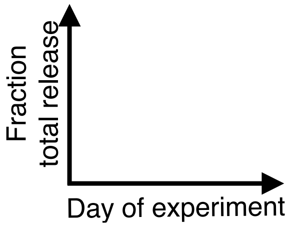

# Human, Treatment **C**

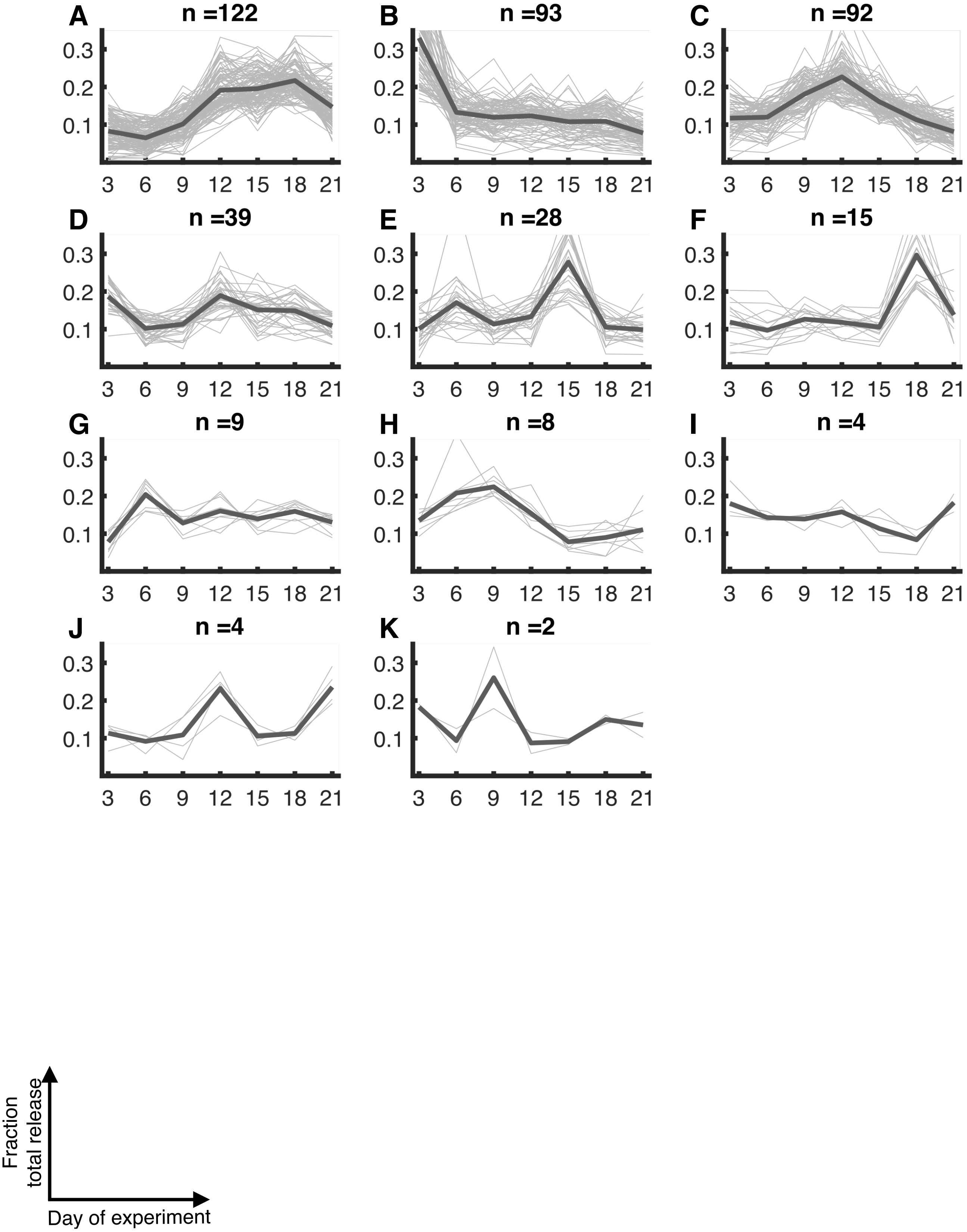

# Human, Treatment **IC**

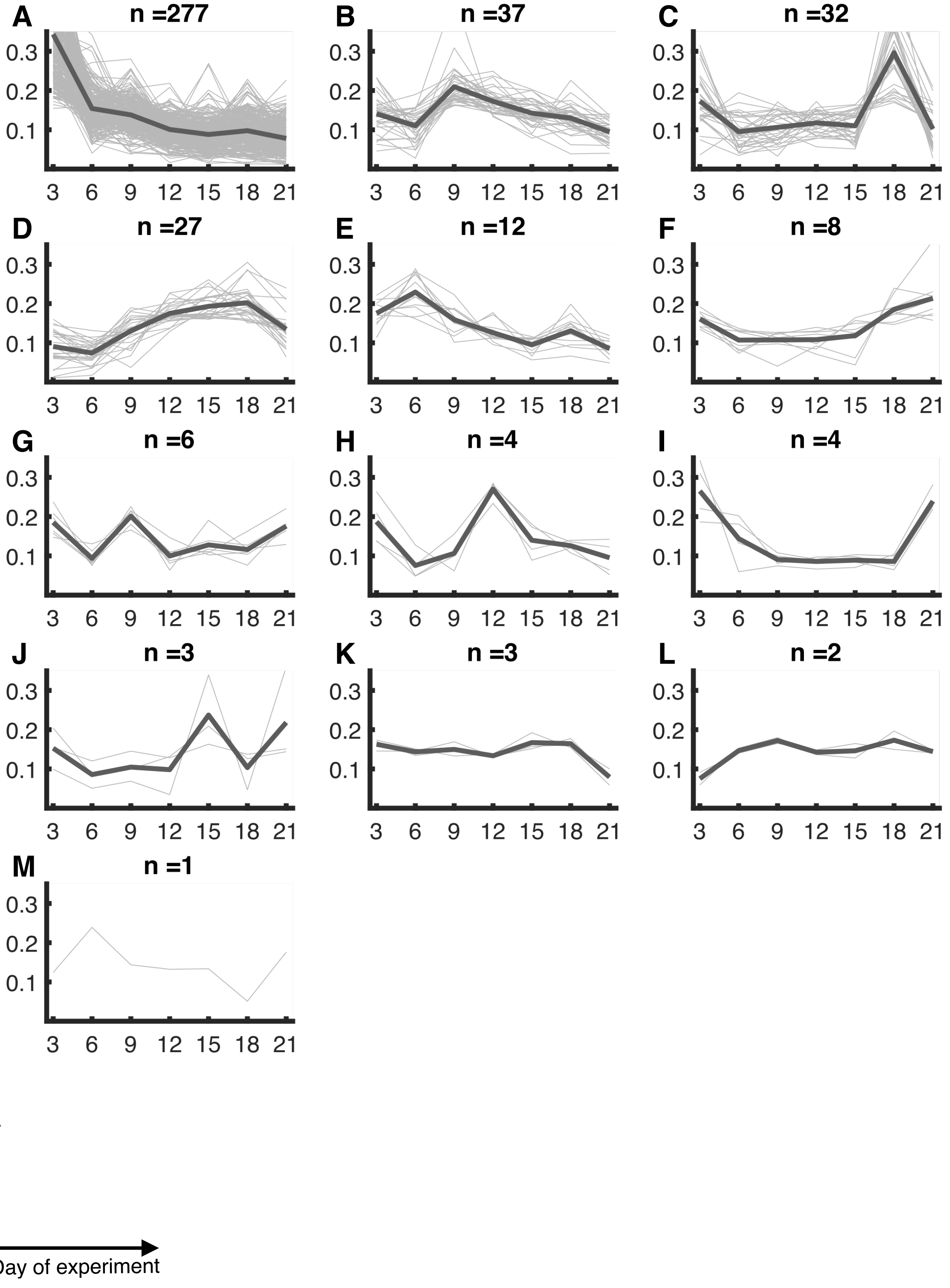

# Human, Treatment **CD**

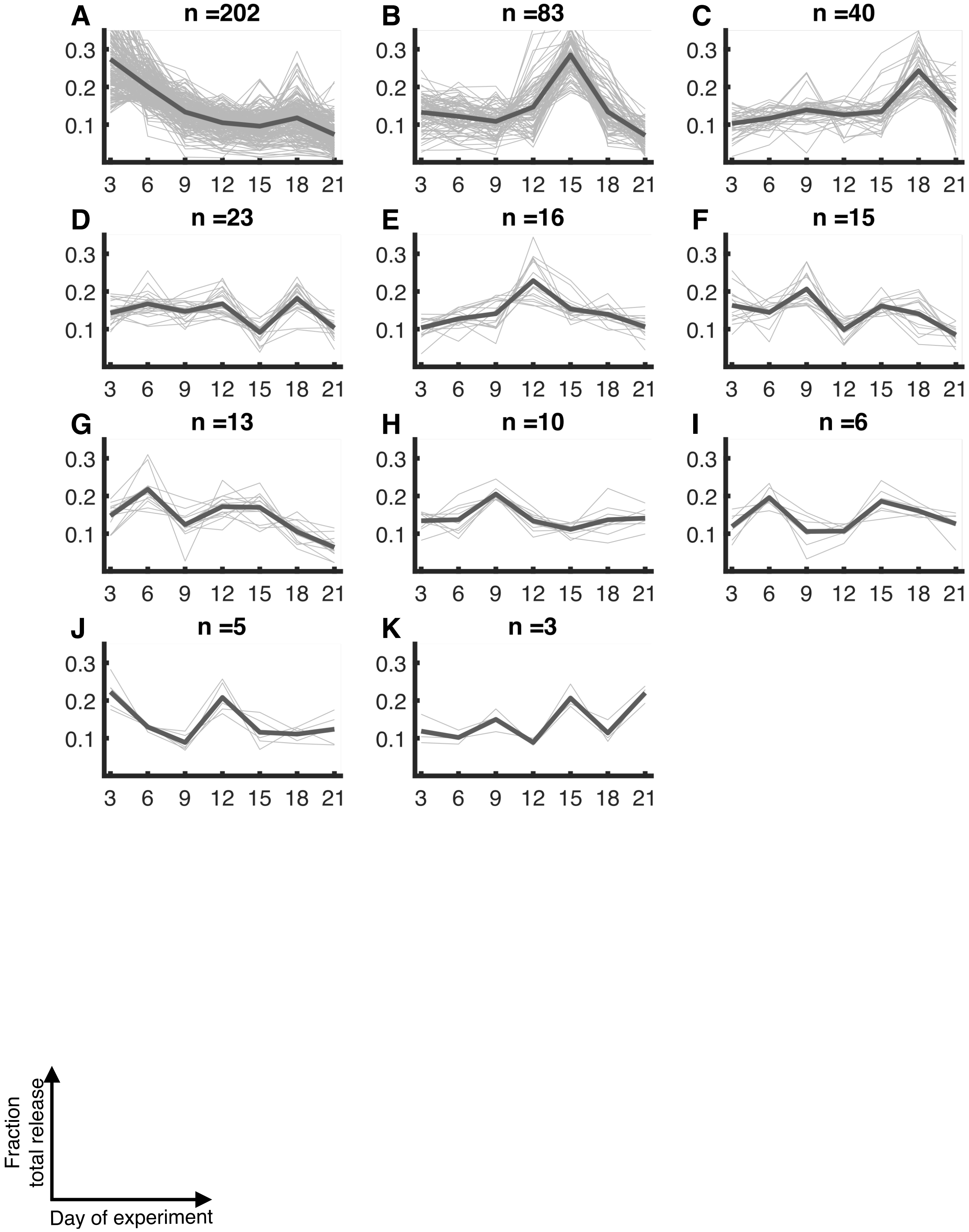

# Human, Treatment **ICD**

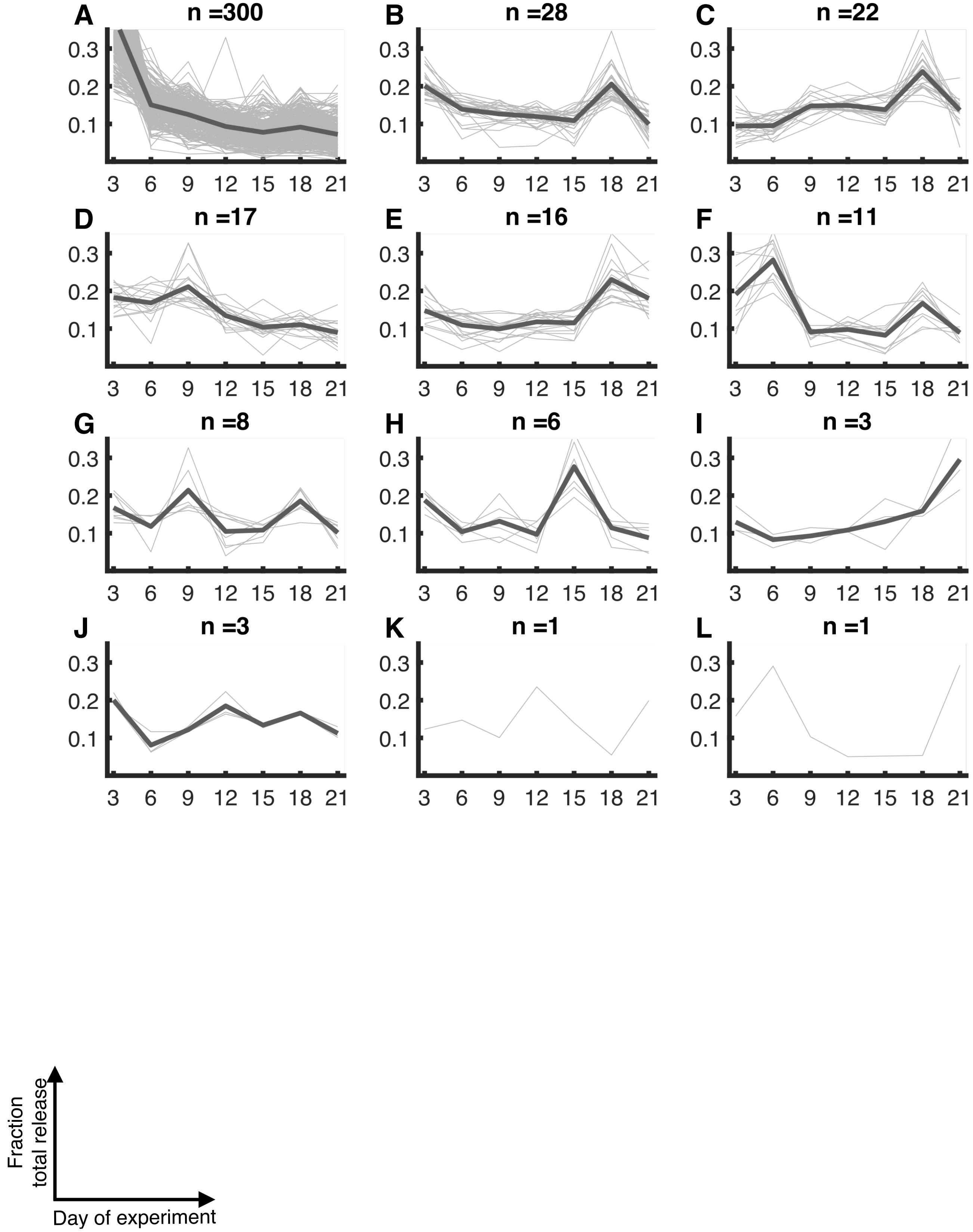

Supplement: Multimedia component 6 [file mmc6.pdf]
