# Supplementary figures and images for: Proteomic clustering reveals the kinetics of disease biomarkers in bovine and human models of post-traumatic osteoarthritis
Source: Osteoarthr Cartil Open. 2021 Jun 10;3(4):100191. doi: 10.1016/j.ocarto.2021.100191 (PMC9611763; doi:10.1016/j.ocarto.2021.100191)

# Bovine: selected proteins from Fig. 5

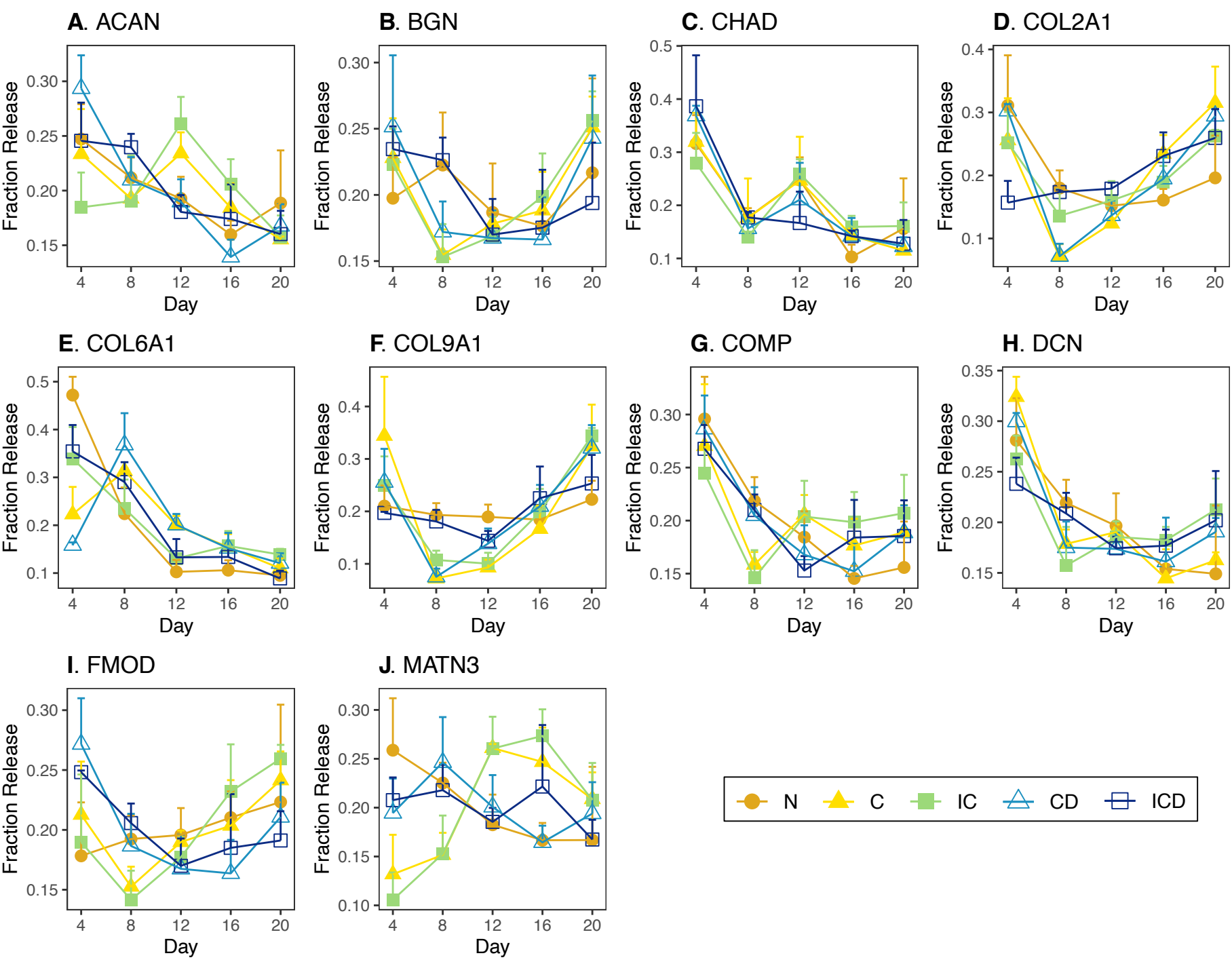

# Human: selected proteins from Fig. 6

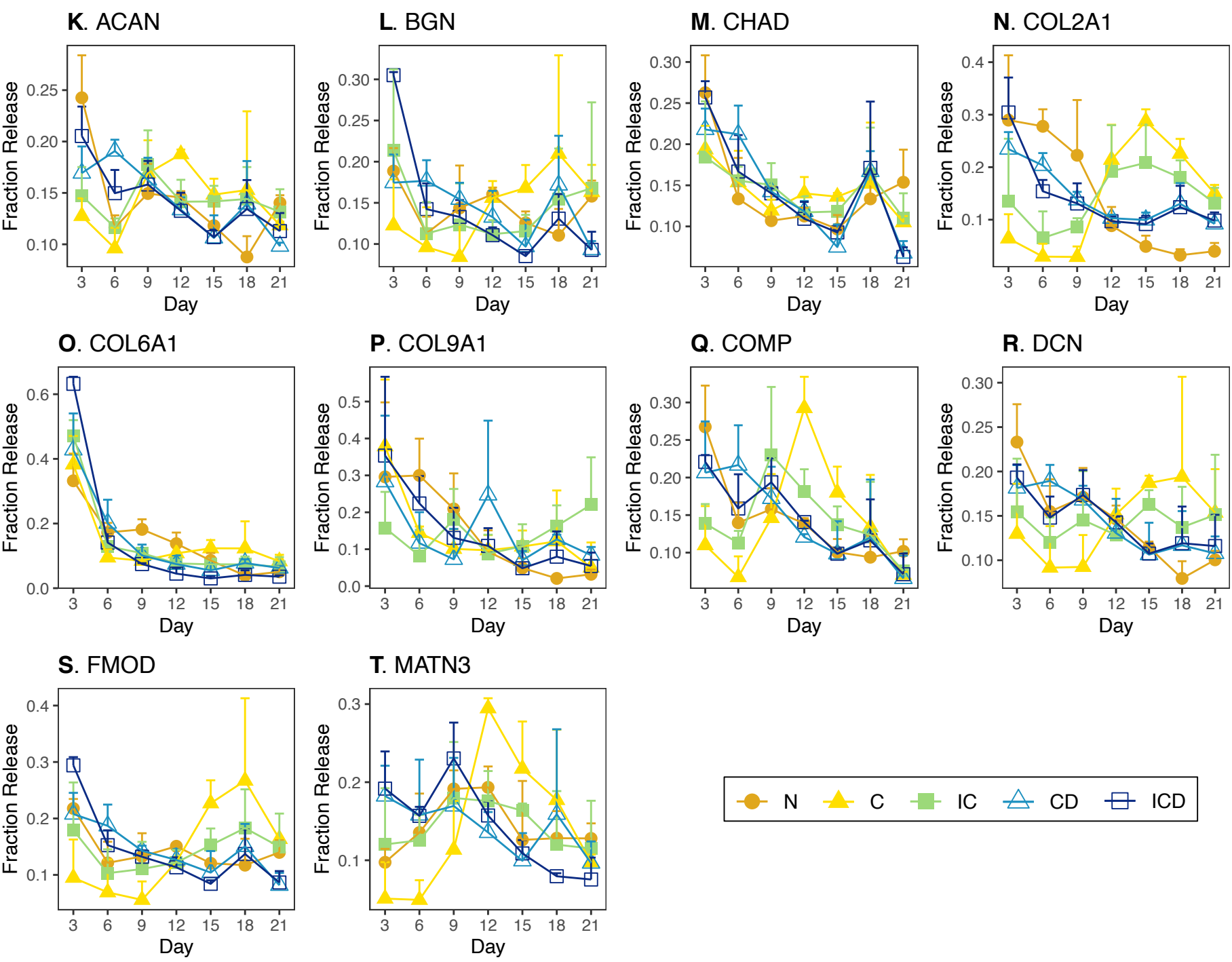

Supplement: Multimedia component 8 [file mmc8.pdf]
