# Supplementary material for: Proteomic clustering reveals the kinetics of disease biomarkers in bovine and human models of post-traumatic osteoarthritis
Source: Osteoarthr Cartil Open. 2021 Jun 10;3(4):100191. doi: 10.1016/j.ocarto.2021.100191 (PMC9611763; doi:10.1016/j.ocarto.2021.100191)
Supplement: Multimedia component 9 [file mmc9.pdf]

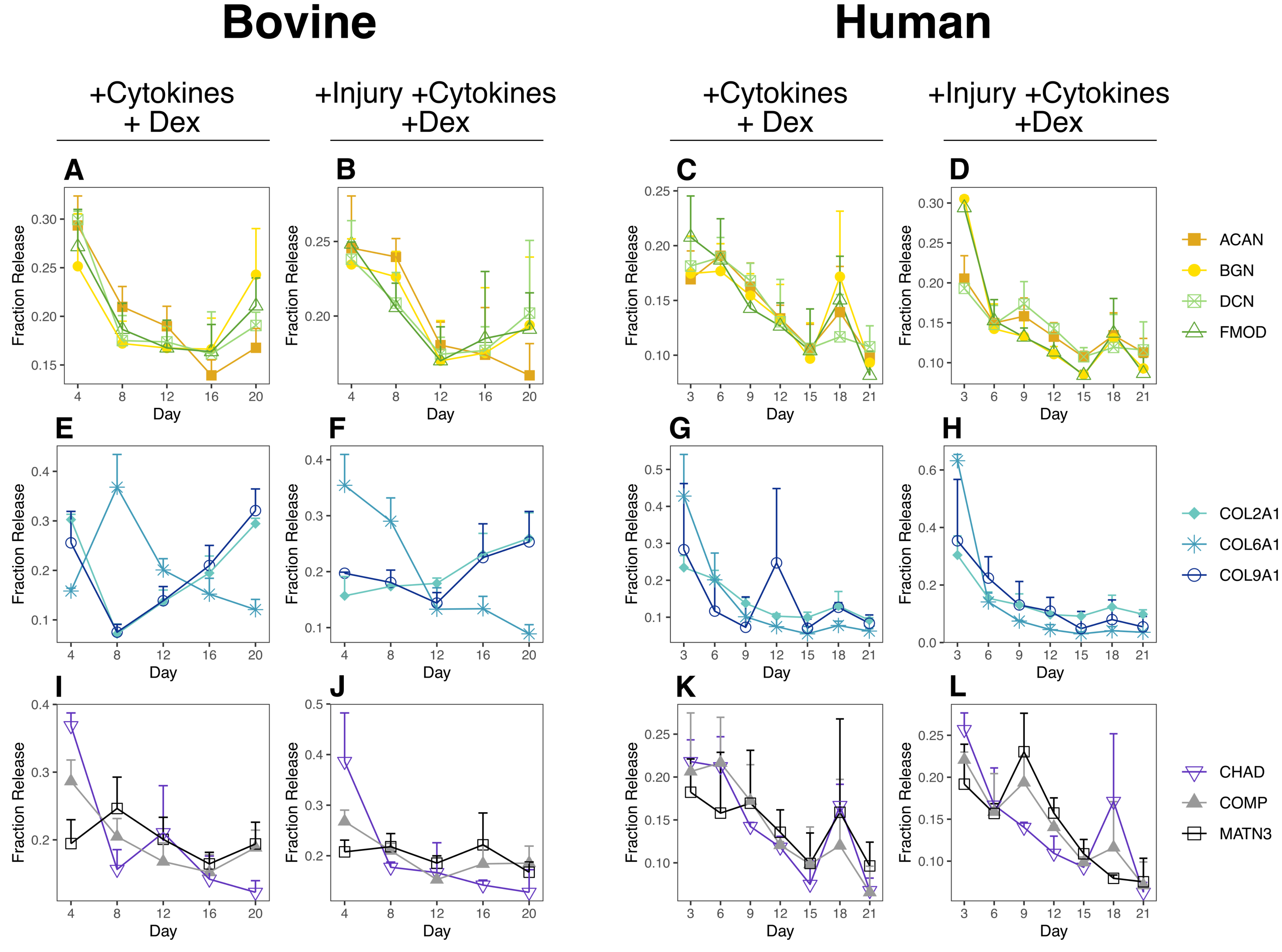
Bovine
Human
+Cytokines + Dex
+Injury +Cytokines +Dex
+Cytokines + Dex
+Injury +Cytokines +Dex
A
B
C
D
E
F
G
H
I
J
K
L
Fraction Release
Day
ACAN
BGN
DCN
FMOD
COL2A1
COL6A1
COL9A1
CHAD
COMP
MATN3
